# Shielding Effects Provide a Dominant Mechanism in J-Aggregation-Induced Photoluminescence Enhancement of Carbon Nanotubes

**DOI:** 10.1021/acsomega.4c00240

**Published:** 2024-03-26

**Authors:** Hubert Piwoński, Kacper Szczepski, Mariusz Jaremko, Łukasz Jaremko, Satoshi Habuchi

**Affiliations:** Biological and Environmental Science and Engineering Division, King Abdullah University of Science and Technology, Thuwal 23955-6900, Saudi Arabia

## Abstract

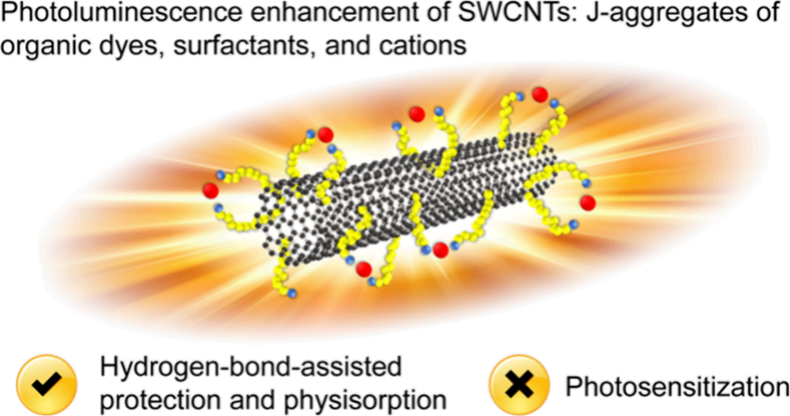

The unique photophysical properties of single-walled carbon nanotubes (SWCNTs) exhibit great potential for bioimaging applications. This led to extensive exploration of photosensitization methods to improve their faint shortwave infrared (SWIR) photoluminescence. Here, we report the mechanisms of SWCNT-assisted J-aggregation of cyanine dyes and the associated photoluminescence enhancement of SWCNTs in the SWIR spectral region. Surprisingly, we found that excitation energy transfer between the cyanine dyes and SWCNTs makes a negligible contribution to the overall photoluminescence enhancement. Instead, the shielding of SWCNTs from the surrounding water molecules through hydrogen bond-assisted macromolecular reorganization of ionic surfactants triggered by counterions and the physisorption of the dye molecules on the side walls of SWCNTs play a primary role in the photoluminescence enhancement of SWCNTs. We observed 2 orders of magnitude photoluminescence enhancement of SWCNTs by optimizing these factors. Our findings suggest that the proper shielding of SWCNTs is the critical factor for their photoluminescence enhancement, which has important implications for their application as imaging agents in biological settings.

## Introduction

The composition of sp^2^-hybridized carbon can be tailored to meet the desired structural performance of nanomaterials of different dimensionalities, including graphene, fullerene, and single-walled carbon nanotubes (SWCNTs). Their exceptional intrinsic properties allowed broad application in diverse areas, including energy storage, chemical processing, quantum photonics, optoelectronics, bioimaging, biotechnology, and medicine.^1−3^ To harness their unique properties in applications to biological systems, one must convert them from organic- to water-soluble and conquer the tendency to agglomerate into large aggregates or bundles before their implementation to any practical use. Even the least invasive surface modification of carbon nanomaterials can impact the intrinsic properties since their constituent atoms reside entirely on the surface. Consequently, surface chemistry is the most essential part of tailoring their properties. Noncovalent functionalization helps in improving solubility while preserving the intrinsic properties of the system. Thus, noncovalent surface coating by aromatic compounds, amphiphilic molecules, polymers, and DNA has become the most common approach for generating stable and biocompatible aqueous dispersions of different carbon nanomaterials.

Among those nanomaterials, SWCNTs have attracted tremendous attention as unique nanoscale shortwave infrared (SWIR) light emitters of high photostability, making them promising candidates for optoelectronics, deep-tissue imaging,^4^ and sensing applications.^5^ Their optical bandgap and distinct electronic properties determined by the quasi-1D structure with a highly delocalized π-electron network result in narrow and diameter-dependent optical bands, allowing for the tuning of absorption and emission by controlling the nanotube diameter and chirality during the synthesis or postsynthetic sorting.^6−10^ SWCNTs have been successfully utilized as fluorescence probes, including hyperspectral imaging in live mammalian cells,^11^ probing cell surface receptors,^12^ subcellular localization in plant cells,^13^ deep-tissue imaging,^14,15^ and whole-body small animal imaging.^16,17^ Although SWCNTs display stable photoluminescence, they typically show low fluorescence quantum yield (QY) in an aqueous suspension (QY varies between 10^–4^ and 0.01),^14,18,19^ significantly limiting their single-particle deep-tissue imaging and tracking applications.^20^ Therefore, developing methods to enhance the photoluminescence brightness of SWCNTs is crucial for improving their sensitivity and detection capabilities.

Various factors contribute to the quenching of the photoluminescence of SWCNTs, including sonication conditions,^21^ electron-transfer reactions,^22^ presence of transition-metal ions,^23^ hole doping,^24^ and chemically disrupted sp^2^-hybridized carbon lattice.^25−28^ Minimizing those effects by attenuating nonradiative relaxation processes leads to QY enhancement,^14,29−31^ resulting in improved photoluminescence brightness. Mild covalent functionalization,^30,32−37^ surface coating by ionic surfactants,^38−41^ ssDNA,^42^ and defect passivation^43,44^ have shown significant effects on the photoluminescence enhancement of SWCNTs. In aqueous dispersion, QY as high as QY = 0.08 has been reported for individual SWCNTs (e.g., not an ensemble average).^45^

Photosensitization (e.g., excitation energy transfer (EET) from photosensitizers to SWCNTs) is an alternative approach to enhance the photoluminescence of SWCNTs. Photosensitizing molecules could be encapsulated in SWCNTs^46,47^ or attached to the side walls of SWCNTs by either van der Waals, π–π, or charge-transfer interactions.^26,48−53^ Since the light absorption of the sensitizers and following EET to SWCNTs determine the photoluminescence enhancement, a significant enhancement is, in principle, achieved by depositing a sensitizer with a large absorption cross section at a high density on the surface of SWCNTs. Recently, photoluminescence enhancement of SWCNTs by a molecular aggregate of organic dyes with a slip-stacked arrangement (i.e., J-aggregate) assembled on SWCNTs has been reported.^54−56^ J-aggregates are unique fluorescent supramolecular assemblies formed by highly ordered organic dyes characterized by a narrow absorption band, large enhanced absorption cross section, and fast and coherent exciton delocalization and migration.^57,58^ Thus, in principle, J-aggregate-SWCNT complexes could provide highly emissive nanocomposites, allowing imaging experiments under challenging conditions (e.g., single-particle imaging in highly scattering and autofluorescing environments). However, due to their complicated excited-state dynamics and interactions with surrounding environments (e.g., ionic surfactants, counterions, and SWCNTs), the exact mechanisms of photosensitizer-mediated photoluminescence enhancement of SWCNTs, in particular J-aggregates-induced photoluminescence enhancement, remain elusive.

**Figure 1:**
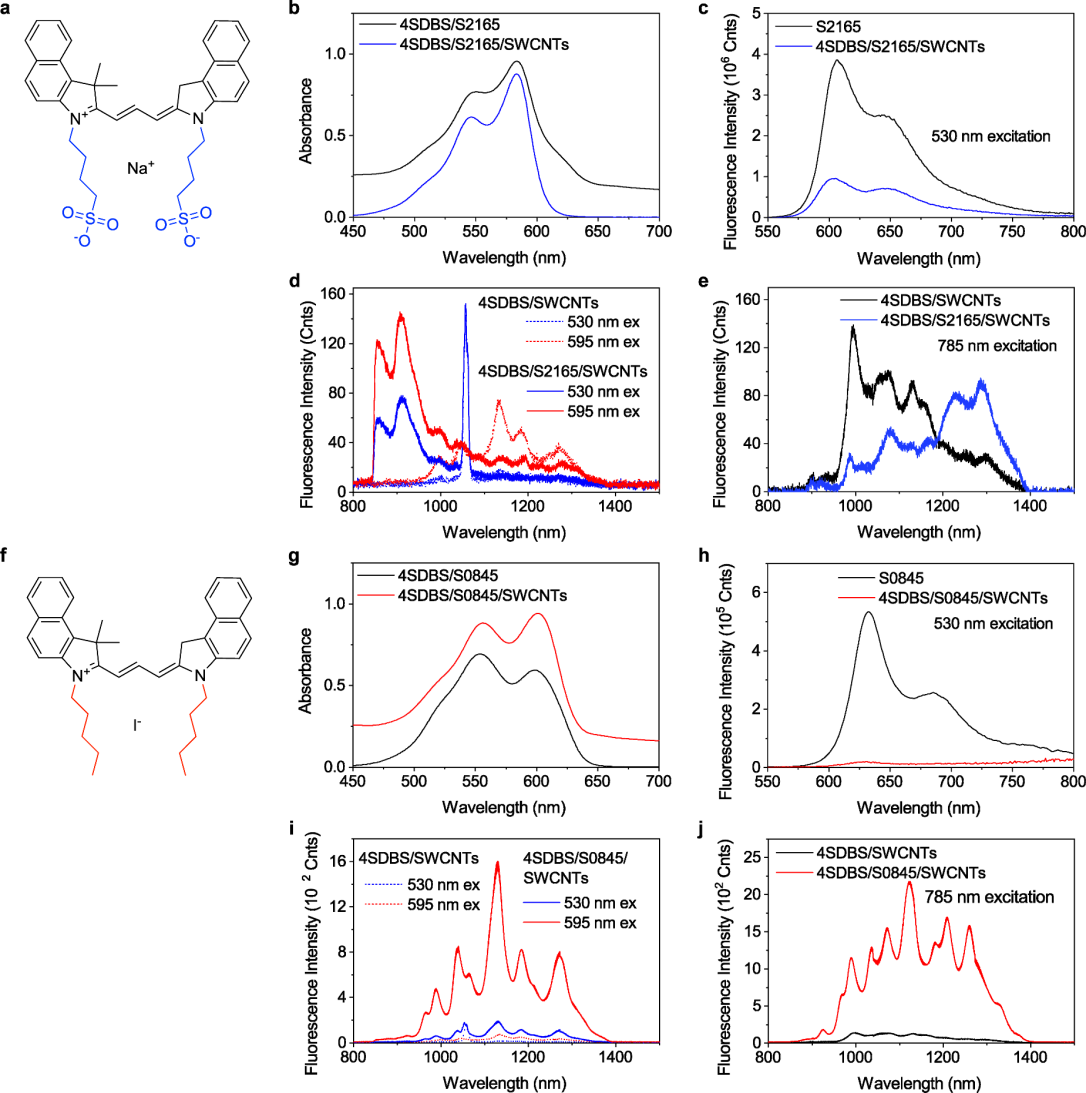
(a) Chemical structure of water-soluble S2165 cyanine dye containing sulfobutyl chains. (b) Absorption spectra of S2165 in water-4SDBS (black) and in the presence of 4SDBS-stabilized HiPco SWCNTs (blue). (c) Fluorescence spectra of S2165 in water-4SDBS (black) and in aggregated form in the presence of 4SDBS-stabilized HiPco SWCNTs (blue) upon 530 nm excitation. (d) SWIR fluorescence spectra of 4SDBS-stabilized HiPco SWCNTs in the absence (dashed lines) and presence (solid lines) of S2165 upon 530 nm (blue) and 595 nm (red) excitation. The sharp peaks observed at 1060 nm are the scattering of the excitation light. (e) SWIR photoluminescence spectra of 4SDBS-stabilized HiPco SWCNTs (black) and the same nanotubes in the presence of S2165 (blue) upon 785 nm excitation. (f) Chemical structure of the solvent-insoluble S0845 cyanine dye with propenyl chains. (g) Absorption spectra of S0845 dispersed in water-4SDBS (black) and in the presence of 4SDBS-stabilized HiPco SWCNTs (red). (h) Fluorescence spectra of the S0845 aggregates in water-4SDBS (black) and in the presence of 4SDBS-stabilized HiPco SWCNTs (red) upon 530 nm excitation. (i) SWIR photoluminescence spectra of 4SDBS-stabilized HiPco SWCNTs in the absence (dashed lines) and presence (solid lines) of S0845 recorded upon 530 nm (blue) and 595 nm (red) excitation. (j) SWIR photoluminescence spectra of 4SDBS-stabilized HiPco SWCNTs (black) and the same nanotubes in the presence of S0845 (red) recorded upon excitation at 785 nm.

In this study, we investigated detailed mechanisms of SWCNT-assisted J-aggregate formation using two cyanine dyes (S2165 and S0845, Figure 1) with different water solubilities and associated photoluminescence enhancement of SWCNTs. We found that the photoluminescence intensity of SWCNTs was enhanced about a hundredfold under optimized conditions. In addition, narrow absorption bands of the J-aggregates on SWCNTs allowed us to separate the impact of environmental factors and EET on the photoluminescence enhancement of the J-aggregate-SWCNT complexes. Surprisingly, our findings suggest a dominant contribution of the shielding effect of the SWCNTs on the photoluminescence enhancement with a negligible contribution of EET.

## Results and Discussion

### Spectroscopic Properties of Cyanine Dye-SWCNT Complexes

The water-soluble cyanine dye S2165 (Figure 1a) in water-4SDBS solution has a maximum absorption at 583 nm with a corresponding sub-band at 547 nm, showing intense emission spectra peaking at 606 nm (Figure 1b,c). The introduction of S2165 into an aqueous dispersion of HiPco SWCNTs stabilized by a surfactant, sodium 4-dodecylbenzenesulfonate (4SDBS), did not affect the peak absorption of S2165 (Figure 1b). However, this led to spectral broadening (Figure 1b), indicating the interaction between S2165 and the SWCNTs. We observed a significant quenching of S2165 fluorescence upon mixing with the 4SDBS-stabilized HiPco SWCNTs (Figure 1c), also indicating their interaction and associated changes in the excited-state processes of S2165. A weak SWIR photoluminescence of 4SDBS-stabilized HiPco SWCNTs was observed upon excitation at 530 or 595 nm (Figure 1d), whereas the photoluminescence spectra of the S2165-SWCNT complex in the SWIR spectral region excited at 530 or 595 nm were dominated by the tail fluorescence of S2165 (Figure 1d) without any detectable photoluminescence of SWCNTs. The result does not support photosensitization by the adsorbed S2165 dyes. SWIR photoluminescence of the 4SDBS-stabilized HiPco SWCNTs in the water excited at 785 nm (i.e., outside the spectral range of the S2165 absorption) was partially quenched in the spectral range 950–1200 nm upon adding S2165 (Figure 1e). This is accompanied by the concomitant slight fluorescence enhancement in the spectral range of SWCNTs with large diameters (Figure 1e), indicating substantial variations among the different nanotubes.

The solvent-soluble cyanine dye S0845 (Figure 1f) in a water-4SDBS solution exhibited a peak absorption at 553 nm accompanied by a less intense vibronic band at 597 nm (Figure 1g). The change in the absorption spectrum compared with that in an organic solvent (methanol) indicates the formation of S0845 aggregates in the water-4SDBS solution (Figure S1). S0845 in water-4SDBS showed a less bright fluorescence emission upon excitation at 530 nm than S2165, with a maximum located at 632 nm (Figure 1h). We observed a change in the absorption spectra of S0845 upon adding it to an aqueous dispersion of the 4SDBS-stabilized HiPco SWCNTs, including a more intense bathochromic peak at 596 nm and a sub-band at 557 nm (Figure 1g and Figure S2a), which indicates the interaction of S0845 and the SWCNTs. Mixing S0845 and the 4SDBS-stabilized HiPco SWCNTs resulted in almost complete quenching of the S0845 fluorescence upon excitation at 530 nm (Figure 1h and Figure S2b). We also observed an enhancement in the photoluminescence brightness of the 4SDBS-stabilized HiPco SWCNTs excited at 530 or 595 nm in the presence of S0845 (Figure 1d,i). While the result potentially indicates efficient photosensitization through EET from the excited state of S0845 to the SWCNTs, we also found that the photoluminescence of the 4SDBS-stabilized HiPco SWCNTs in water excited at 785 nm was enhanced by a factor of 15 upon adding S0845 (Figure 1j and Figure S2c,d). The result may indicate that the photoluminescence of SWCNTs could be enhanced by surface-adsorbed dye molecules through a mechanism different from photosensitization.

### Photoluminescence Enhancement of SWCNTs through J-Aggregate Formation

**Figure 2:**
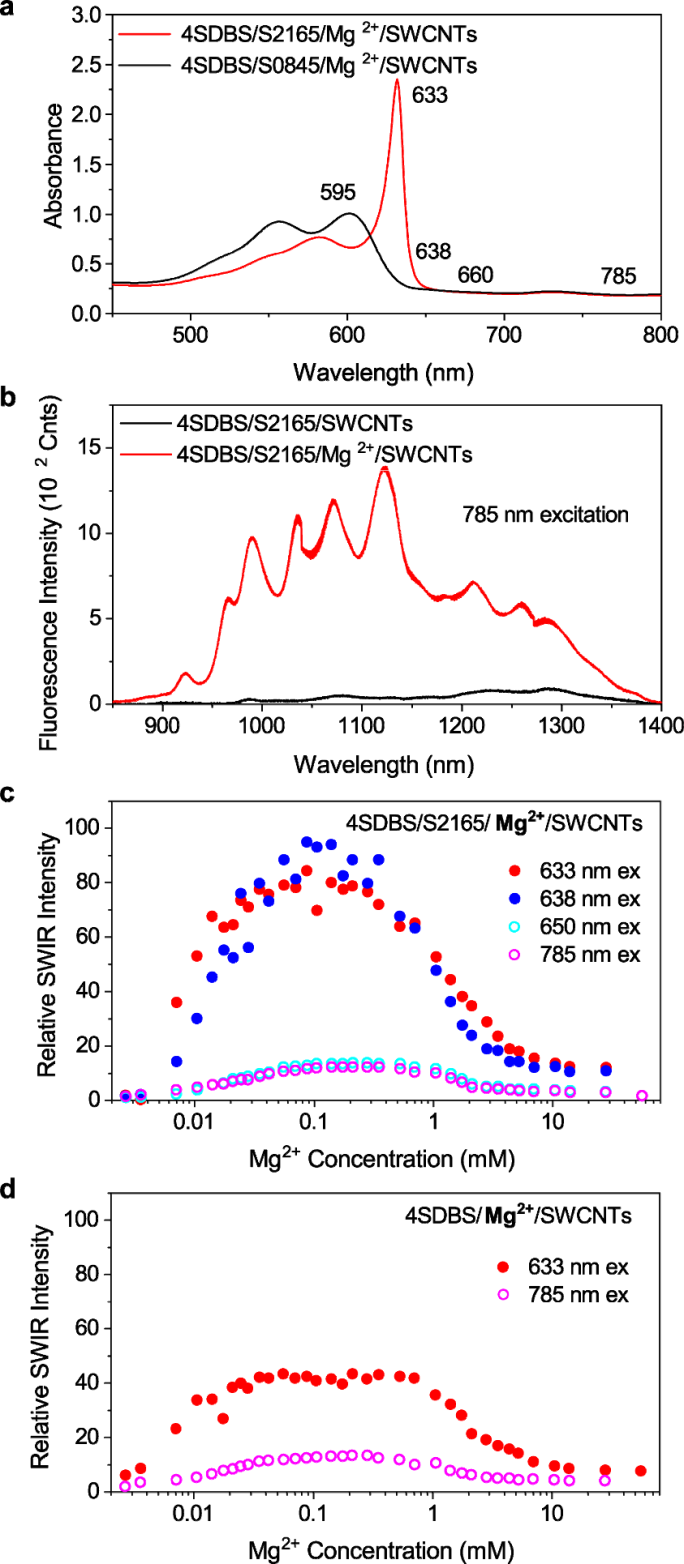
(a) Normalized absorption spectra of the S2165 J-aggregates (red) and S0845 aggregates (blue) on the 4SDBS-stabilized HiPco SWCNTs with the depicted excitation wavelength used in the titration experiment shown in panels (c) and (d). Absorption spectra of 4SDBS-stabilized HiPco SWCNTs dispersed in water were used as a reference to remove the contribution of the absorption bands of SWCNTs. (b) SWIR photoluminescence spectra of the S2165-4SDBS-stabilized HiPco SWCNT complex in the absence (blue) and presence (red) of 35 μM Mg^2+^ upon 785 nm excitation. (c) Relative SWIR photoluminescence intensities of the S2165-4SDBS-stabilized HiPco SWCNT complex at varied concentrations of MgCl_2_ upon excitation at 633 nm (J-band, red), 638 nm (outside the J-band, blue), 650 nm (cyan), and 785 nm (magenta). (d) Relative SWIR photoluminescence intensities of the 4SDBS-stabilized HiPco SWCNT complex at varied concentrations of MgCl_2_ upon excitation at 633 nm (red) and 785 nm (magenta). Each point in panels (c) and (d) represents the integrated photoluminescence of the SWCNT dispersion at a specific titrant concentration divided by the integrated photoluminescence of intact/untreated SWCNT dispersion.

A previous study on a cyanine dye structurally similar to S2165 showed J-aggregate formation on 4SDBS-stabilized HiPco SWCNTs.^54^ In our experiment, we did not observe the formation of J-aggregates of S2165 upon mixing with the 4SDBS-stabilized HiPco SWCNTs in water. We thus added cation (Mg^2+^) to the SWCNT suspension, which promotes the J-aggregate formation of organic dyes in aqueous solution.^59,60^ We found the appearance of a sharp red-shifted absorption band at 632 nm, a signature of the formation of S2165 J-aggregates, upon mixing the dye with the 4SDBS-stabilized HiPco SWCNT aqueous suspension in the presence of a low concentration of MgCl_2_ (Figure 2a and Figure S3a). We note that the concentration of MgCl_2_ required for the formation of the S2165 J-aggregates on the 4SDBS-stabilized HiPco SWCNTs (up to 1.75 × 10^–4^ M, Figure S3a) is much lower than that required for the S2165 J-aggregate formation in an aqueous solution (up to 1 M, Figure S4). In addition, the peak absorption of the S2165 J-aggregates formed on the 4SDBS-stabilized HiPco SWCNTs (633 nm, Figure 2a and Figure S3a) is much shorter than those formed in water at elevated MgCl_2_ concentrations (640 nm, Figure S4), demonstrating SWCNT-assisted formation of the J-aggregates (i.e., not salt-induced J-aggregation).

Concomitant with the appearance of the red-shifted sharp absorption band, the fluorescence of S2165 showed progressive intensity reduction with the appearance of a characteristic persistent deep cut in the spectra, which perfectly overlaps with the peak absorption of the newly appearing J-band (Figure S3b). This sharp dip is attributed to the reabsorption of the monomer S2165 fluorescence by the J-aggregates formed on the SWCNTs, with an efficient EET from the excited state of the S2165 J-aggregates to the nanotube scaffold.

We found that the photoluminescence of the S2165-4SDBS-stabilized HiPco SWCNT complex excited at 785 nm (i.e., direct excitation of SWCNTs) enhanced significantly (15-fold) upon adding MgCl_2_ (Figure 2b, and S3c). The significant photoluminescence enhancement of the 4SDBS-stabilized HiPco SWCNTs by the S2165 J-aggregates (Figure 2b, Figure S3c) upon excitation at 785 nm (i.e., the wavelength with no absorption of the adsorbed dyes) prompted us to perform a more in-depth investigation of the involvement of EET from the S2165 J-aggregates to the SWCNTs in the photoluminescence enhancement of SWCNTs. To that end, we utilized the narrow absorption band of the S2165 J-aggregates in the S2165-SWCNT complex. We excited the complex at four excitation wavelengths: 633 nm for the selective excitation of the S2165 J-aggregates and 638, 660, and 785 nm for the selective excitation of the SWCNTs (Figure 2a). The formation of the S2165 J-aggregates by adding MgCl_2_ resulted in up to nearly 2 orders of magnitude enhancement in the SWIR emission of the S2165-SWCNT complex when excited at 633 nm (Figure 2c). Importantly, we did not observe any significant difference in the photoluminescence enhancement of the S2165-SWCNT complex between 633 and 638 nm excitation by adding MgCl_2_ (Figure 2c), although there is a substantial difference in the excitation efficiency of the S2165 J-aggregates in these two wavelengths. This result raises doubts about the involvement of EET (i.e., photosensitization) in the photoluminescence enhancement of SWCNTs by the adsorbed dye molecules, which has been reported by many previous studies.^49−52,54^

We found that the photoluminescence of the 4SDBS-stabilized HiPco SWCNTs excited at 633 nm was enhanced up to 45 times by adding MgCl_2_ (Figure 2d). This result strongly suggests that EET from the S2165 J-aggregates to the SWCNTs is not the primary mechanism for the enhancement of the SWCNT photoluminescence, and a different mechanism is dominant in the observed photoluminescence enhancement of the SWCNTs. The photoluminescence enhancement of the S2165-SWCNT complex up to 15-fold was observed at 650 and 785 nm excitation by adding MgCl_2_ (Figure 2d). A similar photoluminescence enhancement was observed for the 4SDBS-stabilized HiPco SWCNTs excited at 785 nm by the addition of MgCl_2_ (Figure 2d). Given that there is no light absorption of S2165 at these wavelengths, these results further suggest that the photoluminescence of SWCNTs could be enhanced by a mechanism different from photosensitization.

Interestingly, the adsorption of S0845 to the 4SDBS-stabilized HiPco SWCNTs led to photoluminescence enhancement of SWCNTs similar to that induced by Mg^2+^, including their wavelength dependence (i.e., 45- and 15-fold enhancement when excited at 595–633 and 785 nm, respectively, Figure S 2d), although their (photo)physical interaction with SWCNTs must be very different. We also found that the J-aggregation of S2165 on the 4SDBS-stabilized HiPco SWCNTs led to the marginal photoluminescence enhancement of SWCNTs compared with S0845 and Mg^2+^ when excited at 633 nm (Figure 2d and Figure S 2d). In addition, their contributions to the photoluminescence enhancement of SWCNTs excited at 785 nm are nearly identical (Figure 2c,d and Figure S 2d). The fact that S2165 and S0845 interact with SWCNTs differently (i.e., electrostatic interaction and hydrophobic interaction for S2165 and S0845, respectively, see Supporting Text 1) may indicate that a shielding effect (i.e., physical isolation of SWCNTs from the aqueous solution) is dominant in the observed photoluminescence enhancement of SWCNTs. The observed general trend (i.e., wavelength-dependent photoluminescence enhancement of SWCNTs by S2165, S0845, and Mg^2+^) could also be explained by the diameter (curvature)-dependent coverage of SWCNTs by adsorbed molecules reported previously (see Supporting Text 2).^61,62^ The excess 4SDBS in the S2165-SWCNT complex suspension led to up to 20-fold enhancement of SWCNT photoluminescence when excited at 785 nm (see Supporting Information, Text 3), further supporting this hypothesis.

### Contribution of the Photosensitization on the Photoluminescence Enhancement of SWCNTs

**Figure 3:**
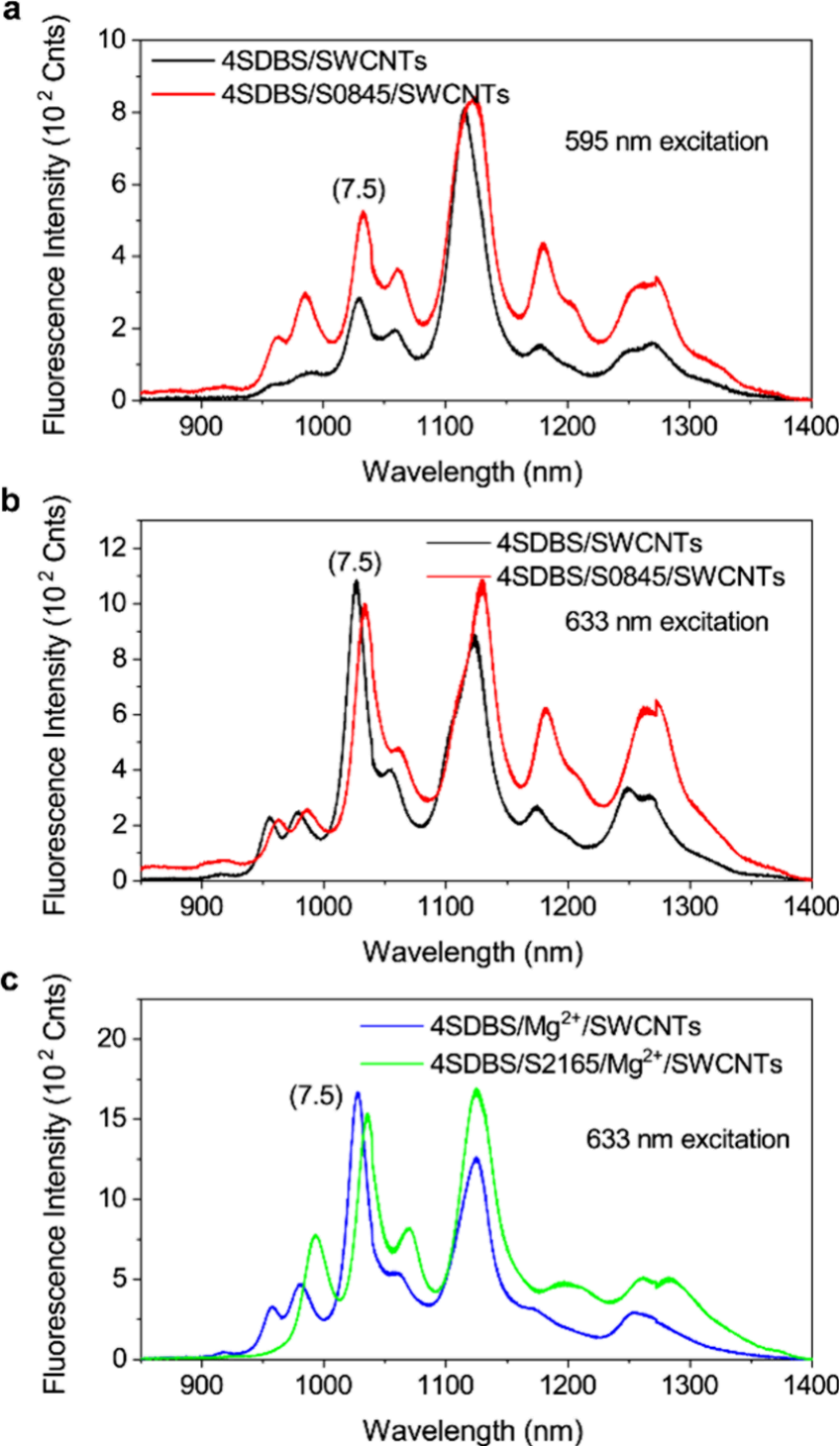
SWIR photoluminescence spectra of 4SDBS-stabilized HiPco SWCNTs in water (black) and in an aqueous solution containing 10.8 μM S0845 (red) upon excitation at (a) 595 and (b) 633 nm. (c) SWIR photoluminescence spectra of 4SDBS-stabilized HiPco SWCNTs in an aqueous dispersion containing 210 μM Mg^2+^ (blue) and the S2165-4SDBS-stabilized HiPco SWCNT complex in an aqueous dispersion containing 105 μM Mg^2+^ (green). The spectra were recorded upon 633 nm excitation. The position of the emission peak corresponding to (7.5) chiral nanotubes has been indicated in all panels.

Photosensitization (i.e., EET from adsorbed dyes to SWCNTs) has been reported as a primary origin of the photoluminescence enhancement of dye-coated SWCNTs.^48,51,52,54,63,64^ However, our results showed that adsorbed dyes, surfactants, and cations affect the photoluminescence behavior of SWCNTs, questioning the contribution of EET to the dye-induced photoluminescence enhancement of SWCNTs. Nearly complete quenching of the S0845 fluorescence upon mixing with the 4SDBS-stabilized HiPco SWCNTs (Figure 1h) may still indicate the existence of EET. Thus, we compared the photoluminescence spectra of the 4SDBS-stabilized HiPco SWCNTs and the 4SDBS-stabilized HiPco SWCNTs mixed with S0845. If EET-induced photoluminescence enhancement occurs, then the (7.5) chirality of SWCNTs that have absorption at a fluorescence wavelength of S0845 would be selectively enhanced. Photoluminescence spectra of 4SDBS-stabilized HiPco SWCNTs mixed with S0845 upon excitation at 595 nm (i.e., the absorption band of S0845) revealed overall 2-fold enhancement as compared to 4SDBS-stabilized HiPco SWCNTs, including the emission peak corresponding to (7.5) chiral nanotubes (Figure 3a), which may be attributed to EET.^54^ However, photoluminescence spectra of SWCNTs mixed with S0845 recorded upon 633 nm excitation (out of the dye absorption range) revealed a further increase in the (7.5) SWCNT emission peak (Figure 3b), which cannot be explained by the EET mechanism. In addition, we did not observe a significant difference in the (7.5) emission peak in the photoluminescence spectra of 4SDBS-stabilized HiPco SWCNTs and those mixed with S0845 upon 633 nm excitation (Figure 3b). Together, these results suggest that the contribution of EET to the photoluminescence enhancement of the SWCNTs is marginal, although their involvement cannot be entirely excluded.

We also found that the 4SDBS-stabilized HiPco SWCNTs and the 4SDBS-stabilized HiPco SWCNTs mixed with S2165 in the presence of Mg^2+^ (i.e., S2165 J-aggregates-SWCNT complex) showed very similar photoluminescence spectra (Figure 3c). Although the presence of both S2165 J-aggregates and monomers on SWCNTs makes the quantitative discussion difficult, the observation is consistent with the conclusions derived from the S0845-SWCNT complexes.

### Contribution of the Shielding Effect on the Photoluminescence Enhancement of SWCNTs

The shielding effect is a potential alternative mechanism for the photoluminescence enhancement of SWCNTs by the 4SDBS surfactant, Mg^2+^, and cyanine dyes. It is well-known that surfactant–SWCNT interactions modify the characteristics of SWCNTs. Although the surface coating of SWCNTs by surfactants is primarily for improving its dispersion stability in an aqueous environment and the separation of SWCNTs with different chirality,^65,66^ the surface coating by surfactants has been reported to contribute to the enhancement of the photoluminescence of SWCNTs.^14^ Previous studies suggested that 4SDBS adsorbs on SWCNTs in two steps.^67−69^ The first step occurring at a low concentration is random adsorption of 4SDBS, with their hydrophobic tails parallel to the long axis of SWCNTs.^67^ At a high concentration, 4SDBS is reorganized into a more ordered adsorption with their aliphatic tails arranged perpendicular to the long axis of SWCNTs.^67^ The observed 4SDBS concentration-dependent photoluminescence enhancement (Figure S5) indicates that SWCNTs are better protected from the environment when 4SDBS has tightly packed ordered adsorption on SWCNTs.

**Figure 4:**
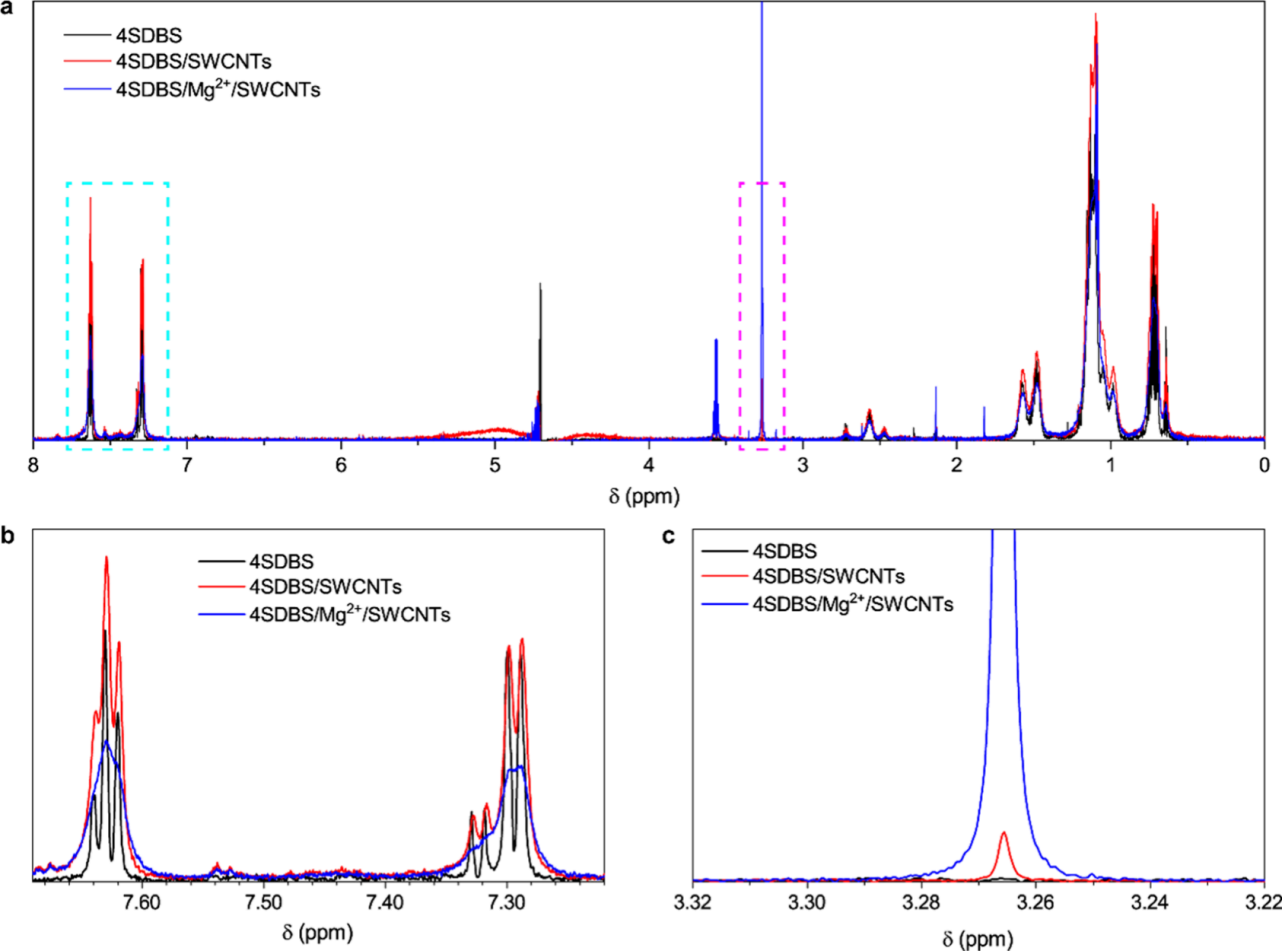
(a) 1D ^1^H NMR spectra of free 4SDBS in water (black) and 4SDBS adsorbed on the surface of HiPco SWCNTs in the absence (red) and presence (blue) of Mg^2+^. Enlarged views of the spectra are highlighted by (b) cyan rectangle and (c) magenta rectangle in (a).

The addition of cations to surfactant-coated SWCNTs was reported to have an additional protection effect on SWCNTs, leading to the photoluminescence enhancement of SWCNTs,^8,40^ although the mechanisms of the photoluminescence enhancement have not been fully characterized. Previous studies indicated that cations might penetrate the hydrogen shell of the surfactant head groups and restrict the mobility of water molecules.^70^ Cations were also reported to trigger the reorganization of the surfactants into a tight and ordered configuration.^8,41,71−73^ This could contribute to the photoluminescence enhancement of the SWCNTs. To test this hypothesis, we performed a 1D ^1^H NMR study on 4SDBS-stabilized HiPco SWCNTs dispersions in the absence and presence of Mg^2+^ and compared them with a ^1^H NMR spectrum of a free 4SDBS in water. A significant change in ^1^H NMR spectra of 4SDBS was observed at the 7–8 ppm range, which can be assigned to aromatic protons of the benzenesulfonate group, and at 3.26 ppm, which is not associated with any protons of the 4SDBS molecule structure (Figure 4 and Figure S6). Signals from the aromatic protons of 4SDBS in water appeared in the form of triplet and doublet of doublets around 7.62 and 7.30 ppm, respectively, while lacking any signal at 3.26 ppm (Figure 4b,c). In the presence of SWCNTs, the triplet and doublet of doublets broadened and reduced their intensities (Figure 4b). Simultaneously, a weak signal at 3.26 ppm appeared (Figure 4c). The broadening of the proton signals from the benzenesulfonate group is interpreted by a partially restricted mobility of the protons in the presence of SWCNTs. Upon adding Mg^2+^, the signals from the aromatic protons merged and further decreased their intensity due to increased restriction of the mobility of protons, reflecting the rearrangement of the benzenesulfonate groups and the formation of the bridged configuration with Mg^2+^. Simultaneously, the peak at 3.26 ppm increased massively.

Computer simulation studies indicated that Mg^2+^ could enter into the first hydration shell of the head groups of sulfonate surfactants, cause the ordering of water molecules around the head groups, and increase the strength of hydrogen bonds.^74,75^ These studies also indicated that water molecules in the first hydration shell bind to the sulfonate group either directly or through bridging by introduced Mg^2+^. Our 1D ^1^H NMR experiment provides the first direct evidence that a new hydrogen bonding network formed between water molecules and 4SDBS on the surface of SWCNTs is significantly enhanced by Mg^2+^ (see Supporting Text 4). This finding strongly suggests that the observed Mg^2+^-induced photoluminescence enhancement of the 4SDBS-stabilized SWCNTs by a factor of up to 45 can be interpreted by the hydrogen bond-assisted isolation of SWCNTs from the bulk water molecules, a well-known fluorescence quencher.^76,77^

### Salt-Induced Reorganization of Cyanine Dyes Interacting with 4SDBS-Stabilized SWCNTs and Its Contribution to the Photoluminescence Enhancement of SWCNTs

**Figure 5:**
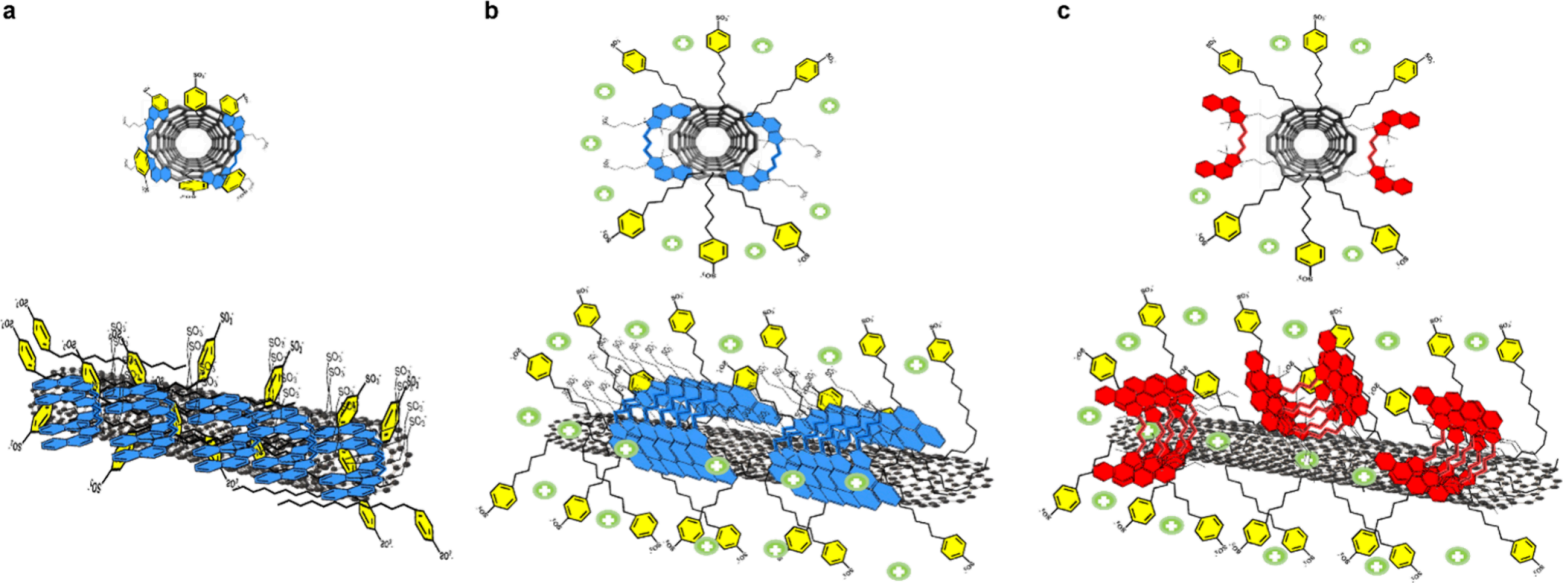
Proposed model of S2165 dye aggregation on 4SDBS-stabilized HiPco SWCNTs (a) in the absence of Mg^2+^ and (b) in the presence of Mg^2+^. (c) Model of the S0845 dye aggregation on the 4SDBS-stabilized HiPco SWCNTs.

The salt-induced J-aggregation of S2165 and its contribution to the photoluminescence enhancement of SWCNTs are also accounted for by the salt-induced reorganization of 4SDBS. The fluorescence of S2165 and the photoluminescence of the 4SDBS-stabilized SWCNTs are both quenched upon their interaction (Figure 1c–e). A similar mutual quenching was reported for a cationic organic dye, methylene blue, which was attributed to a charge-transfer complex with an in-plane orientation of the dyes on the surface of SWCNTs.^61,78^ This indicates that S2165 interacts with the 4SDBS-stabilized SWCNTs with an in-plane orientation due to the electrostatic attraction between the positively charged chromophore unit and the highly negatively charged SWCNT surface by 4SDBS (Figure 5a). Adding Mg^2+^ to the suspension led to the significant enhancement of the photoluminescence of SWCNTs (2 orders of magnitude enhancement, Figure 2c). As discussed above, most of the observed enhancement (up to 45-fold) can be attributed to the reorientation of 4SDBS and associated hydrogen bond-assisted bridging of 4SDBS by Mg^2+^ (see Supporting Text 5).

This salt-induced surfactant reorganization causes the reorientation of adsorbed S2165 to an out-of-plane orientation through the similar bridging effect between the sulfonic groups of benzenesulfonate and the sulfonyl groups of the sulfobutyl chains of S2165, which is suitable for the formation of J-type aggregates (Figure 5b). In this configuration, the chromophores remain close to the surface of SWCNTs by the electrostatic attraction between the positive charge on the chromophore and the negatively charged surface of SWCNTs. At the same time, the aliphatic chains containing sulfonic groups are exposed to the solution phase and stabilized by Mg^2+^ bridging with benzenesulfonate groups of 4SDBS. Therefore, the further protection of SWCNTs by the S2165 J-aggregates accounts for the twofold enhancement of the photoluminescence of SWCNTs. The photoluminescence enhancement of 4SDBS-stabilized SWCNTs by the adsorption of S0845 (Figure S 2d) suggests an out-of-plane orientation of S0845 (Figure 5c). In this configuration, S0845 attaches to the surface of the SWCNTs through the aliphatic chains by hydrophobic interactions. The positively charged S0845 chromophore remains exposed to the solution phase and stabilized by interacting with negatively charged benzosulfonic surfactant groups in the chromophore-bridged configuration. The minor enhancement of the photoluminescence of SWCNTs upon adding Mg^2+^ indicates insignificant reorganization of S0845 and 4SDBS upon adding the salt (Figure S7). The S0845 fluorescence remains quenched on the 4SDBS-stabilized SWCNTs, suggesting an efficient EET to SWCNTs due to its proximity to the SWCNT surface. Together, our findings demonstrate that the photoluminescence enhancement induced by the surface-adsorbed cyanine dyes is predominantly caused by the shielding effect of the SWCNTs from the surrounding bulk water molecules.

### Effect of the Surface Curvature

The data presented in this study, as well as those in the previous studies, suggest that 4SDBS on SWCNTs reorganize from the random configuration with their tails parallel to the SWCNT’s long axis into a more ordered configuration with their tails arranged perpendicular to the SWCNT's long axis upon increasing the surfactant concentration or adding electrolytes. Since SWCNTs with smaller curvature (i.e., SWCNTs with larger diameters that emit photoluminescence at longer wavelengths)^79^ are coated by the surfactant molecules more efficiently,^80,81^ the surfactant coverage-induced reorganization of 4SDBS to the perpendicular configuration would occur much efficiently at a given 4SDBS concentration. Indeed, we observed chirality-dependent photoluminescence enhancement of SWCNTs with a gradual increase in the 4SDBS concentration in the SWCNTs dispersion (Figure S8).

**Figure 6:**
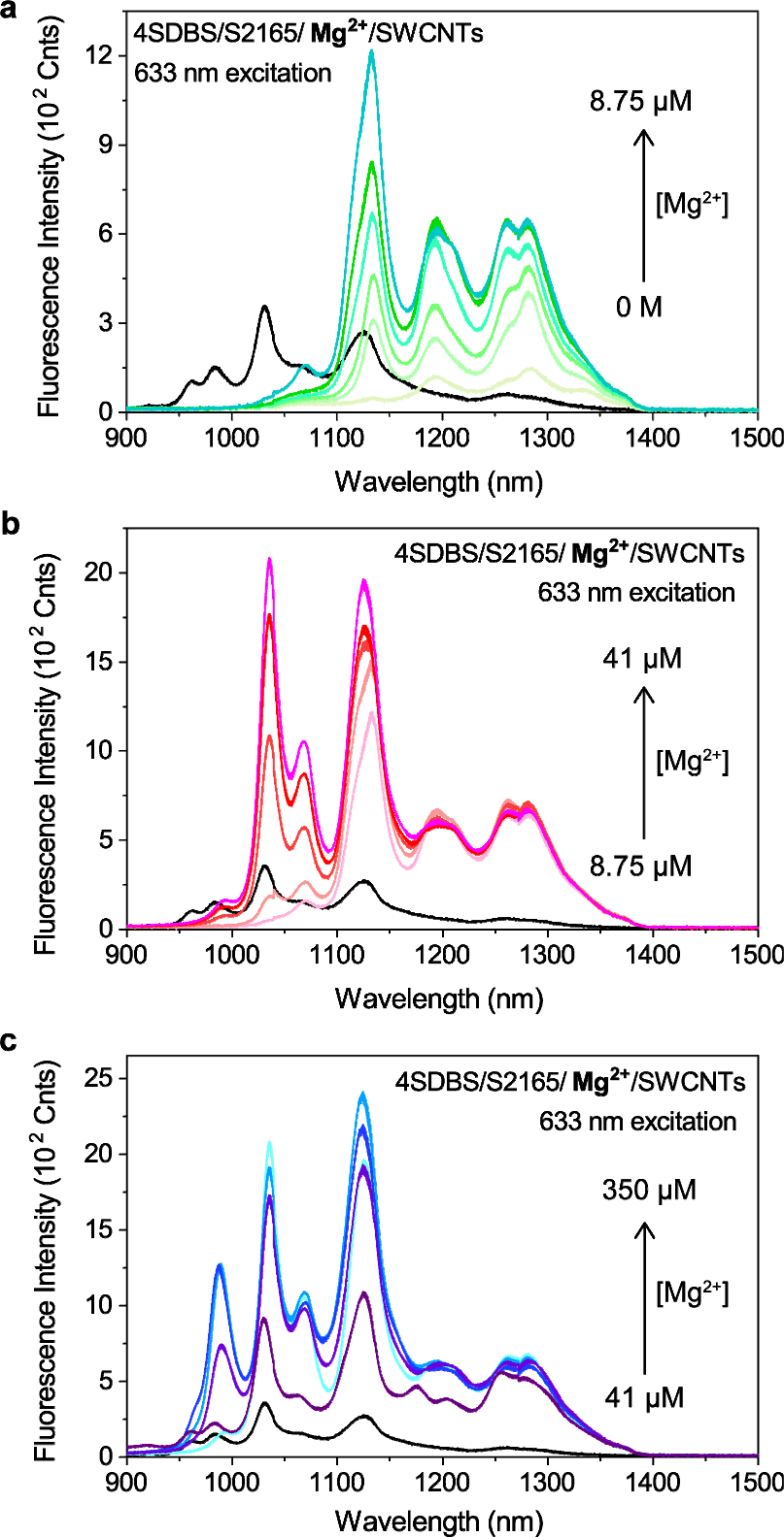
Spectrally resolved curvature-dependent enhancement in the SWIR photoluminescence of 4SDBS-stabilized HiPco SWCNTs mixed with S2165 at varied concentrations of MgCl_2_. (a) 0–8.75 μM, (b) 8.75–41 μM, and (c) 41 μM–0.35 mM Mg^2+^. Spectra were recorded upon 633 nm excitation. The absorption spectra of the S2165-SWCNT complex in the absence of Mg^2+^ are also included in panels (b) and (c) (black lines) as a reference.

Similarly, since the salt-bridged perpendicular configuration of 4SDBS is essential for the formation of the Mg^2+^-induced S2165 J-aggregates on SWCNTs, this Mg^2+^-induced process would be much more efficient for SWCNTs with a smaller curvature at a given Mg^2+^ concentration. Indeed, we found selective photoluminescence enhancement of SWCNTs with smaller curvatures in the S2165-SWCNT dispersion exposed to low Mg^2+^ concentrations (Figure 6a). At higher Mg^2+^ concentrations, the photoluminescence of SWCNTs with larger curvatures that emit at shorter wavelengths was also enhanced (Figure 6b,c), confirming 4SDBS reorganization and S2165 J-aggregation in the salt-bridged perpendicular configuration. These results demonstrate the curvature-dependent protection of SWCNTs by the surfactant and cyanine dye, which leads to the curvature-dependent enhancement of the photoluminescence of SWCNTs. It is also indicated that the characteristic dimensionality of SWCNTs (i.e., a flat surface along the long axis and a curved surface along the short axis) enables the reorganization of 4SDBS and, therefore, the salt-induced photoluminescence enhancement.

**Figure 7:**
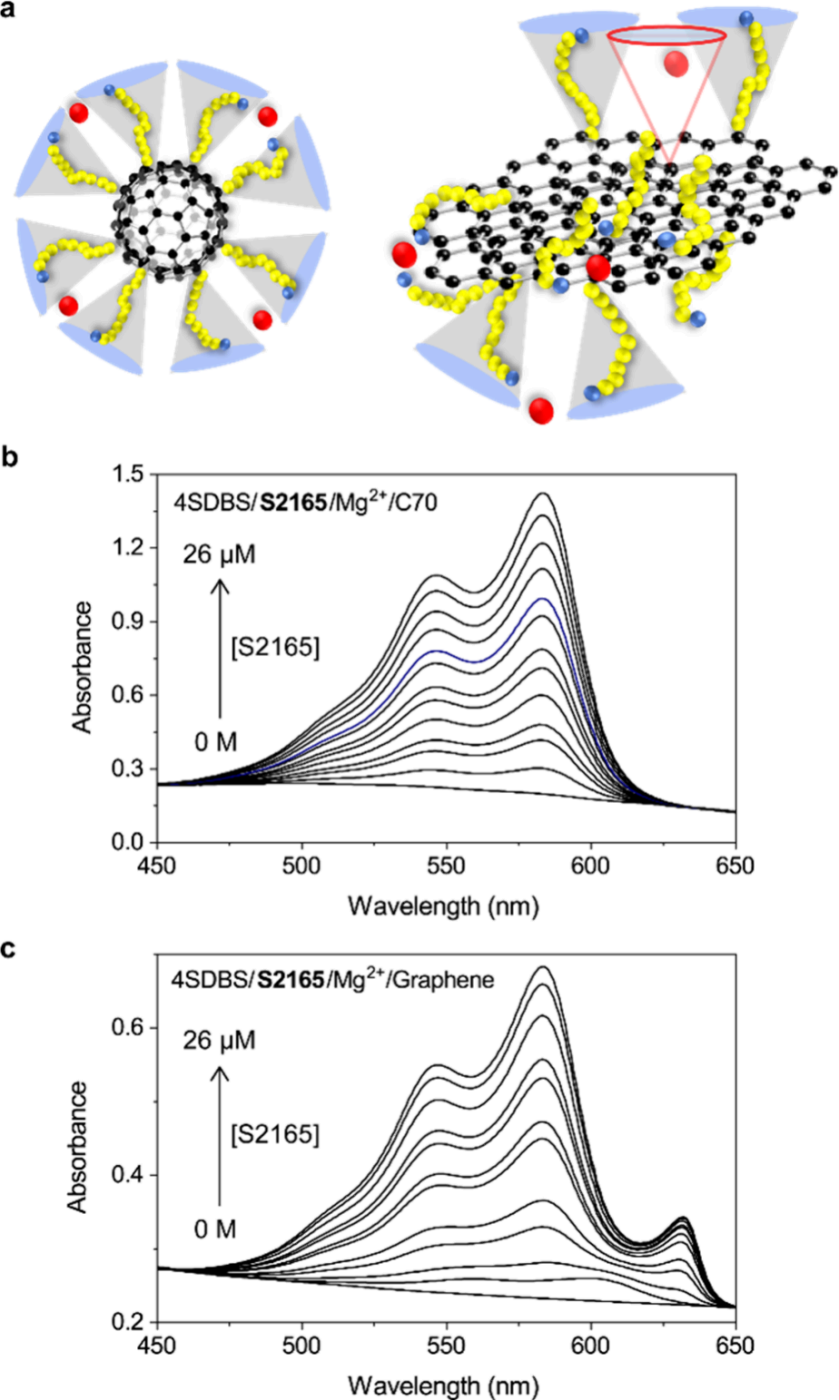
Effect of the surface curvature of carbon nanomaterials on the rearrangement of 4SDBS surfactant and associated S2165 J-aggregation. (a) Model of 4SDBS arrangement on C70 fullerene (left) and graphene (right) nanoparticles in the presence of Mg^2+^. (b) Absorption spectra of the S2165–C70 fullerene composite at varied concentrations of S2165 (0–26 μM) with 175 μM Mg^2+^. (c) Absorption spectra of the S2165–graphene composite at varied concentrations of S2165 (0–26 μM) with 175 μM Mg^2+^.

In addition, a lack of chiroptical activity of the S2165 J-aggregates-SWCNT complex in the circular dichroism experiment indicates the absence of the torsional arrangement of the S2165 molecules, although such a molecular arrangement cannot be excluded completely due to the racemic nature of SWCNTs (Figure S9). The result may indicate the importance of the flatness along the long axis for the formation of the J-aggregate. We tested this hypothesis by measuring the formation of J-aggregates of S2165 on 4SDBS-stabilized carbon nanomaterials with different dimensionalities, C70 fullerene and graphene (Figure 7a). C70 fullerene has a spherical shape without a flat surface; therefore, the J-aggregation requiring a flat surface would be prevented. Indeed, we did not observe the formation of the S2165 J-aggregates on the surface of the C70 fullerene (Figure 7b). Graphene has a two-dimensional flat shape. While the absence of a curved surface would prevent the efficient formation of the salt-bridged perpendicular configuration of 4SDBS, its flat surface may facilitate the formation of the J-aggregates. As predicted, the S2165 J-aggregates were formed on 4SDBS-stabilized graphene but with relatively low efficiency (Figure 7c). Our results suggest that the curvature and dimensionality play a crucial role in the protection and, thus, the photoluminescence enhancement of SWCNTs by the surfactants, salts, and organic dyes.

## Conclusions

This study investigated the mechanisms responsible for the J-aggregate assembly of cyanine dyes on SWCNTs and associated photoluminescence enhancement. Our findings suggest that surfactant reorganization under the electrolyte perturbation and cation-related bridging arrangement of sulfonic groups of the cyanine dye S2165 and 4SDBS surfactant molecules are responsible for the J-aggregate formation. Importantly, our findings revealed that the excitation energy transfer from the cyanine dyes (i.e., photosensitizer) has a negligible contribution to the photoluminescence enhancement of SWCNTs. Organic molecules are tightly adsorbed on the surfaces of SWCNTs and protect them from water, which accounts for up to a 45-fold enhancement of the photoluminescence of SWCNTs. Similarly, restricting water mobility in the micelle hydration shell by hydrogen bonding with surfactant head groups and the counterions contributes to forming a protective barrier, leading to 45-fold photoluminescence enhancement. Altogether, those mechanisms render almost 2 orders of magnitude photoluminescence enhancement. A more significant protection effect was observed for SWCNTs with smaller diameters, which are the most vulnerable to quenching by water molecules due to the extent of unprotected sidewalls. In addition, this study pointed out that the surface dimensionalities of carbon nanomaterials are crucial for the adsorption and rearrangement of interfacial molecules, including surfactants and small organic dyes. Our findings provide general guidelines for designing and constructing composite nanomaterials based on carbon nanomaterials for imaging applications.

## Experimental Section

SWCNTs RAW HiPco and PureWave graphene with a flake size of 150–200 nm composed of thin 4–7-layer graphene nanoplatelets and average thickness of 2.4 nm were purchased from Integris. Fullerene-C_60_ and [5,6]-fullerene-C70 were purchased from Sigma-Aldrich. Cyanine dyes, S0845 (3-butyl-2-[3-(3-butyl-1,3-dihydro-1,1-dimethyl-2H-benzo[e]indol-2-ylidene)-propenyl]-1-1dimethyl-1H-benzeno[e]indolium iodide) and S2165 (2-[3-[1,1-dimethyl-3-(4-sulfobutyl-1,3-dihydro-1,3-dihydro-benzo[e]indol-2-ylidene]-propenyl]-1,1-dimethyl-3-(4-sulfobutyl)-1H benzo[e]indolium hydroxide, sodium salt), were purchased from FEW Chemicals. 4-Dodecylbenzenesulfonate (4SDBS), sodium chloride, calcium chloride, and magnesium chloride were purchased from Sigma-Aldrich. Lithium acetate dehydrate, silver nitrate, and iron(III) chloride hexahydrate were purchased from Fisher.

SWCNT powder (17 mg) was dispersed in deionized water (50 mL) in the presence of surfactants, e.g., sodium 4-dodecylbenzenesulfonate (4SDBS, 0.4 mM) during 4 h of vigorous sonication using an ultrasonic liquid processor FB705 Sonic Dismembrator (Fisher Scientific) followed by 60 min centrifugation with a 5804 Centrifuge (Eppendorf) at 4200 rpm to remove nondispersed materials (see Supporting Text 6).

Steady-state absorption measurements were performed on a Hitachi U-300 spectrometer (UV-NIR) or a PerkinElmer Lambda-900 spectrometer (UV-SWIR). Steady-state fluorescence measurements were performed on a Horiba FluoroMax-4 spectrofluorometer in the VIS-NIR spectral range or Princeton Instruments IsoPlane spectrograph equipped with PyLoN 1700 InGaAs camera in the SWIR range using either a 785 nm beam of a Ti:saphire laser (Spectra-Physics, MaiTai), Ti:saphire laser (Coherent, Chameleon Ultra) equipped with OPO (Coherent, Chameleon Compact OPO), 632.8 nm line of a HeNe laser (Coherent), or 638 and 660 nm laser diodes (OEM Laser) as excitation sources.^82,83^ We used 1 cm optical path quartz cells (Hellma) for all measurements. The concentration of nanoparticle dispersions in each experiment was adjusted to obtain an optical density of 0.3 at 400 nm (see Supporting Text 7). Typically, 75 μL of 4SDBS stock dispersion was added to 2.925 mL of Milli-Q water, which provided a suspension containing 8.5 μg/mL SWCNTs and 9.6 μM 4SDBS. Circular dichroism spectra were measured on the JASCO J-1500 spectropolarimeter. The concentration of the cyanine dyes was set to 11 μM unless otherwise stated in the figure legends.

NMR samples were prepared by adding 10% (v/v) D_2_O to water-soluble SWCNTs/4SDBS. ^1^H NMR spectra were recorded at 24.85 °C on an 800 MHz Bruker Avance NEO NMR spectrometer equipped with a sensitive triple resonance (H/C/N-D) TCI cryogenic probe. ^1^H 1D data were collected using the standard Bruker pulse program ZGESP with 64 scans and 32,000 data points. NMR data processing and interpretation were performed using Topspin ver. 4.0.7.

## Supporting Information Available

(Supporting Text 1) Binding modes of S2165 and S0845 cyanine dyes to the 4SDBS-stabilized SWCNTs; (Supporting Text 2) effect of the curvatures of SWCNTs on the adsorption of surfactant molecules; (Supporting Text 3) effect of the 4SDBS concentration on the photoluminescence enhancement of SWCNTs; (Supporting Text 4) assignment of 3.26 ppm signal in ^1^HMR spectra to the proton of water; (Supporting Text 5) formation of the Mg^2+^-mediated hydrogen bonding network between 4SDBS on SWCNTs and surrounding water molecules; (Supporting Text 6) bundles of SWCNTs in the samples; and (Supporting Text 7) concentration of the SWCNT suspension, absorption spectra of S0845, absorption and photoluminescence spectra of S0845-SWCNTs complex, effect of Mg^2+^ on the absorption and photoluminescence spectra of the S2165-SWCNT complex, effect of Mg^2+^ on the absorption spectra of S2165, effect of 4SDBS on the absorption and photoluminescence spectra of the S2165-SWCNT complex, ^1^HMR spectra of free 4SDBS and 4SDBS adsorbed on SWCNTs, S0845 concentration-dependent enhancement of SWIR photoluminescence of S0845-SWCNTs complex, 4SDBS concentration-dependent enhancement of SWIR photoluminescence of SWCNTs, circular dichroism spectra of SWCNTs and S2165-SWCNTs complex, effect of Mg^2+^ on the absorption and photoluminescence intensity of the S2165-SWCNT complex, effect of the high concentration of Mg^2+^ on the absorption and photoluminescence spectra of the S2165-SWCNT complex, effect of the concentration and valence of cations on the formation of the S2165 J-aggregates on SWCNTs, absorption spectrum of the S2165-SWCNT complex in D_2_O, SWIR photoluminescence spectra of SWCNTs in H_2_O and D_2_O, and absorption spectrum of 4SDBS-SWCNTs in water (PDF)
