# Supplementary material for: Shielding Effects Provide a Dominant Mechanism in J-Aggregation-Induced Photoluminescence Enhancement of Carbon Nanotubes
Source: ACS Omega. 2024 Mar 26;9(14):16496–507. doi: 10.1021/acsomega.4c00240 (PMC11007775; doi:10.1021/acsomega.4c00240)
Supplement: Supplementary file 1 — ao4c00240_si_001.pdf [file ao4c00240_si_001.pdf]

Supporting Information

Shielding Effects Provide a Dominant Mechanism in J-aggregation-Induced Photoluminescence Enhancement of Carbon Nanotubes

Hubert Piwoński, Kacper Szczepski, Mariusz Jaremko, Łukasz Jaremko, Satoshi Habuchi**

Biological and Environmental Science and Engineering Division, King Abdullah University of Science and Technology, Thuwal 23955-6900, Saudi Arabia

*Correspondence should be addressed to S.H. (email: satoshi.habuchi@kaust.edu.sa) or H.P (email: Hubert.piwonski@kaust.edu.sa).

Contents

Supporting Text: Supporting Text 1 – 7

Supporting Figures: Figures S1 – S15

References

Supporting Text

1. Binding modes of S2165 and S0845 cyanine dyes to the 4SDBS-stabilized SWCNTs.

An excess salt led to reduced J-band intensity, de-quenching of S2165 fluorescence, and reduced SWIR photoluminescence of the 4SDBS-stabilized SWCNTs (Figure S10). The de-quenched fluorescence spectrum of S2165 was red-shifted with the maximum at 618 nm (Figure S11). This indicates that excess salt leads to the dissociation of S2165 aggregates from the surface of 4SDBS-stabilized SWCNTs in the form of new aggregates co-stabilized by the 4SDBS surfactant. Simultaneously, the 4SDBS-stabilized SWCNTs started precipitating from the solution due to the counter-ion-driven screenings of electrostatic repulsion forces and breaking the dispersion.[1] This indicates that the driving force for the interaction between S2165 and 4SDBS-stabilized SWCNTs is generally the Coulombic attraction. Our data suggest that stable S2165 J-aggregates-SWNCTs complex exists only in a specific narrow range of salt concentration (Figure S12). According to the titration curves, the valence and size of the cation determine the efficiency of the J-aggregates formation and its concentration dependence (Figure S12).

On the contrary, adding Mg^{2+} to the S0845-SWCNTs complex led only to slightly enhancing the SWIR photoluminescence (Figure S7). A further increase in the Mg^{2+} concentration resulted in a decrease in SWIR photoluminescence related to breaking the dispersion. However, it did not lead to de-quenching of the fluorescence from the surface-adsorbed S0845 even at the elevated concentrations, indicating the pure hydrophobic nature of the interaction between S0845 and 4SDBS-stabilized SWCNTs.

2. Effect of the curvatures of SWCNTs on the adsorption of surfactant molecules.

The difference in relative photoluminescence enhancement of SWCNTs excited at different wavelengths can be attributed to different chirality subpopulations of SWCNTs preselected at specific excitation conditions (Figure 6, main text). While SWCNTs with smaller diameters have large bandgaps and thus a shorter excitation wavelength, SWCNTs with larger diameters show absorption at a longer wavelength. [2] Each single-chirality species of SWCNTs characterizes different intrinsic brightness.[3, 4] The surfactant coverage is determined by the diameter (i.e., bond curvature) and surface area of SWCNTs.[5] In general, the adsorption capacity of carbon-based nanomaterials decreases as the curvature increases.[6-11] Since higher surfactant coverages impede fluorescence quenching, SWCNTs with larger diameters (lower curvature) that are densely covered by surfactants should be better protected and, thus, reveal relatively small Mg^{2+}-induced photoluminescence enhancement. Indeed, we observed chirality-dependent photoluminescence enhancement of S2165-4SDBS-stabilized HiPco SWCNTs complex in the Mg^{2+} titration experiment (Figure 6, main text). Fluorescence spectra recorded at different Mg^{2+} concentrations indicate the photoluminescence enhancement of selective chirality at specific Mg^{2+} concentrations. Interestingly, the trend follows the order of previously reported SWCNTs chirality-dependent separation on gel chromatography,[12] originating from the fact that SWCNTs with higher surfactant coverage have much weaker interaction with gel and can be collected as a first fraction. The curvature-dependent photoluminescence enhancement observed in this study is consistent with this observation.

3. Effect of the 4SDBS concentration on the photoluminescence enhancement of SWCNTs.

The increase of 4SDBS concentration in the aqueous dispersion of SWCNTs leads to a reduction in the formation of the S2165 J-aggregates (Figure S5a). Since the available surface of 4SDBS-stabilized SWCNTs acts as a scaffold for the J-aggregates formation, the increased surface coverage by 4SDBS results in the reduced S2165 J-aggregates formation. At the same time, less effective quenching of S2165 monomeric fluorescence Figure S5b) and enhanced photoluminescence from the SWCNTs in the examined dispersions were observed (Figure S5c), leading to 20-fold enhancement of the SWIR photoluminescence at excess of 4SDBS.

4. Assignment of 3.26 ppm signal in ^{1}H NMR spectra to the proton of water.

While the chemical shift of the bulk water proton is typically positioned at around 4.7 ppm, the chemical shift of a nuclear spin can be affected by diverse interactions, including anisotropic shielding, hydrogen bonding, electronegativity, and polar effects. Thus, it is expected that the position of the H_2O proton NMR signal varies with respect to the solvent.[13] Indeed, peak shifts of H_2O proton NMR signal (in the range between 3 and 3.4 ppm) due to the change in hydrogen bonding have been utilized to assess the residual water content in organic solvents.[14, 15] A molecular dynamics simulation study indicated that introducing cations into water increases the strength of hydrogen bonds if the cation is located next to the hydrogen-donating water.[16] A simulation study also indicated that all water molecules in the first hydration shell of the sulfonate head group of ionic surfactants are donors for hydrogen bonds,[17] and Mg^{2+} entering the first hydration shell disturbs the hydrogen bonding network, increases hydrogen bond strength, disturbs water orientation, and reduces the number of hydrogen bonds with other water molecules.[17] Under this condition, upfield shifts of the proton NMR signal of water protons bonded to the sulfonate head group are expected by shielding through the oxygen of the sulfonate group. Thus, the 3.26

ppm signal observed in our ^{1}HNMR experiment is assigned to the water in the first hydration layer, forming strong hydrogen bonds with the surfactant head groups. A 3.3 ppm peak in the ^{1}H NMR was observed for SDBS surfactants in DMSO that could be assigned to the residual water interaction with the S=O group of the surfactants through hydrogen bonding,[13, 18] also support our assignment of the 3.26 ppm peak.

5. Formation of the Mg^{2+}-mediated hydrogen-bonding network between 4SDBS on SWCNTs and surrounding water molecules.

The ^{1}H NMR data and a previous molecular dynamics study[19] indicated that the ions strongly affect the hydration structure around the head groups of the surfactant. The Mg^{2+} added to the suspension of 4SDBS-stabilized SWCNTs could screen the repulsion forces between negatively charged sulfonic groups of 4SDBS and reorient the surfactant to a cation-bridged configuration. A previous study also suggested that the local electric field produced by ions could modify the strength of hydrogen bonds.[16] Thus, we tested the hypothesis that the introduction of ions affects the reorganization of surfactant not only by the simple screening of the repulsion forces between the headgroups but also by modifying the strength of hydrogen bonds with interfacial water molecules.[17] To that end, we examined the SWCNTs-assisted J-aggregation of S2165 in D_2O since D_2O is known to have a more ordered structure with stronger hydrogen bonds than water.[20, 21] The introduction of S2165 to 4SDBS-stabilized SWCNTs dispersed in D_2O led to the almost instantaneous formation of J-aggregates without adding Mg^{2+} (Figure S13). According to the model described in the main text, the formation of S2165 J-aggregates requires the reorientation of S2165 on the surface of 4SDBS-stabilized SWCNTs as a response to the surfactant reorganization initiated by counterions. The formation of S2165 J-aggregates in D_2O indicates that

hydrogen bonding is essential in the surfactant reorganization and stabilization of the cation-bridged configuration (through Na^+ that belongs to the surfactant). It is likely that the strong hydrogen bonding in D_2O triggers surfactant reorganization without the perturbation by external electrolytes (e.g., Mg^{2+}), whereas the introduction of external electrolytes is essential for water for modifying hydrogen bonding in the vicinity of the surfactant head group and thus triggering the surfactant reorientation.

This interpretation is partially supported by observed SWIR photoluminescence enhancement of 4SDBS-stabilized SWCNTs in D_2O. Photoluminescence of SWCNTs can be quenched by water molecules in two separate mechanisms. The first mechanism is related to water molecules interacting with the surface of SWCNTs (e.g., defect sites). As discussed in the main text, this quenching mechanism can be eliminated by displacing water from the vicinity of SWCNTs (i.e., the shielding effect through high surface coverage of SWCNTs by the surfactant molecules). The second mechanism is the quenching by high-energy vibrations of -OH moieties of water. In this mechanism, the electronic excitation of SWCNTs is resonantly transferred to high-frequency vibrational stretching modes of water through space, which cannot be eliminated by shielding. This mechanism is commonly observed for organic fluorophores in water,[22-24], leading to a threefold reduction of their fluorescence brightness.[25] Interestingly, the photoluminescence spectra of 4SDBS-stabilized SWCNTs (high 4SDBS coverage) in D_2O revealed a 2.8-fold higher SWIR photoluminescence than 4SDBS-stabilized SWCNTs (high 4SDBS coverage) dispersed in water (Figure S14), which may indicate that both mechanisms contribute to the overall photoluminescence enhancement of 4SDBS-stabilized SWCNTs in D_2O; shielding effect induced by the surfactant reorganization and protecting from resonance electronic energy transfer to overtone OH vibrational modes.

6. Bundles of SWCNTs in the samples.

Centrifugation of aqueous surfactant dispersions is a commonly used technique to separate SWCNTs from bundles and aggregated impurities. In this study, the surfactant-dispersed SWCNTs solution was prepared by vigorously mixing the surfactant and SWCNTs in water under the probe-sonication conditions, which was followed by centrifugation. Previous studies indicated supernatant phase contains the dispersed and mostly exfoliated single SWCNTs. The cryogenic transmission electron microscopy imaging of SWCNT dispersions revealed the presence of both bundled and individual nanotubes when the suspension was not centrifuged. In contrast, only individual nanotubes were observed in the supernatant after centrifugation.[26] Together, although we cannot exclude the possibility of having a small fraction of the bundles in our samples, the contribution of SWCNT bundles would be negligible in our experiments, and thus, the contribution of the bundling to the observed phenomena (e.g., photoluminescence enhancement of SWCNTs) would be minimum.

7. Concentration of the SWCNTs suspension.

We note that we used optical density (OD) at 400 nm (Figure S15) to prepare our SWCNT samples in a consistent manner for the following reasons.

We used OD = 0.3 at 400 nm in combination with mass per volume of the carbon-nanomaterial in the surfactant-water dispersant as a reference point to attain consistent experimental conditions between different carbon-nanomaterial dispersions used in the study. At this wavelength, we have a predominant contribution of the scattering caused by the dispersed carbon nanomaterial. This simplifies the sample preparation and removes the possibility of having artifacts on the data, which helps us to unravel the photophysical and chemical processes reported in this manuscript.

Calculating the "real concentration" of carbon nanotubes in SWCNTs' dispersion using the absorption spectrum and the reported extinction coefficients can be misleading. SWCNT samples are usually mixtures of species possessing different diameters, purity, and chirality types. Since the extinction coefficients of SWCNTs were determined for specific systems, the mass concentration determined by the reported extinction coefficients may not be valid in other systems. The solvent used for the dispersion may also alter the value of the extinction coefficient significantly.

Indeed, the reported extinction coefficients of SWCNTs vary significantly. For example, Landi et al. reported extinction coefficients of 39.0 $mL \cdot mg^{-1} \cdot cm^{-1}$ at 500 nm for SWCNTs in N,N-dimethylacetamide.[27] In other studies on SWCNTs produced by the HiPCo method by different manufacturers and dispersed in 1,2-dichlorobenzene, the extinction coefficients of 28.6 $mL \cdot cm^{-1} \cdot mg^{-1}$ and 43.8 $mL \cdot cm^{-1} \cdot mg^{-1}$ at 500 nm was reported by Bahr et al.[28] and by Ivanova et al.[29] These extinction coefficient values obtained in organic solvents were subsequently used by different groups to analyze SWCNTs dispersions in aqueous solutions.[30-32] Backes, C., et al. reported the extinction coefficient of 11.25 $mL \cdot cm^{-1} \cdot mg^{-1}$ for CoMoCAT SWCNTs at 652 nm.[33]

The varied reported extinction coefficient values lead to different concentrations of SWCNTs in the dispersion. When we use reported values of extinction coefficient (ε) at 500 nm and the formula for optical density $OD = c \cdot l \cdot \varepsilon$, where c is concentration, and l is an optical path (l = 1 cm), the estimated concentration of SWCNTs in our samples varies in the range of 5.4 to 8.3 μg/ml

$c = OD_{500}/(l \cdot \varepsilon) = 0.24/54.2 \approx 5.4$ μg/ml

$c = OD_{500}/(l \cdot \varepsilon) = 0.24/28.6 \approx 8.3$ μg/ml

When we apply the reported extinction coefficient at 652 nm (11.25 $mL \cdot cm^{-1} \cdot mg^{-1}$), the concentration of SWCNTs in our samples is calculated to be:

$c = OD_{652}/(l \cdot \varepsilon) = 0.22/11.25 \approx 2$ μg/ml

Calculating the concentration of SWCNTs based on experimentally determined absorbance at 1030 nm ($\varepsilon_{1030} = 46.9$ mL·cm^{-1}·mg^{-1}) and ($\varepsilon_{660} = 36$ mL·cm^{-1}·mg^{-1}) in N-methyl-2-pyrrolidone (Journal of Nanoscience, 2014, 2014, 328627) leads to the following SWCNTs concentration range:

$c = OD_{1030}/(l \cdot \varepsilon) = 0.16/46.9 \approx 3.4$ μg/ml

$c = OD_{660}/(l \cdot \varepsilon) = 0.20/36 \approx 5.5$ μg/m

The calculation of the molar concentration of SWCNTs is even more complicated as the reported molar extinction coefficients calculated per C-atom differ by order of magnitude.[34] In addition, the conversion to the molar concentration of SWCNTs is affected by the distribution of the length and diameter of the nanotubes in the suspension.

As such, we can only say that the mass concentration of SWCNTs in the suspensions used in this study is in the range of several μg per ml.

Supporting Figures

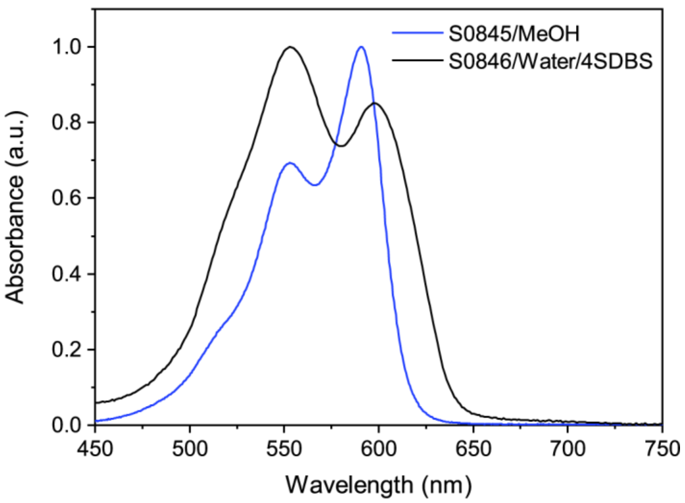

Figure S1. Absorption spectra of S0845 in methanol (blue) and water-4SDBS solution (black).

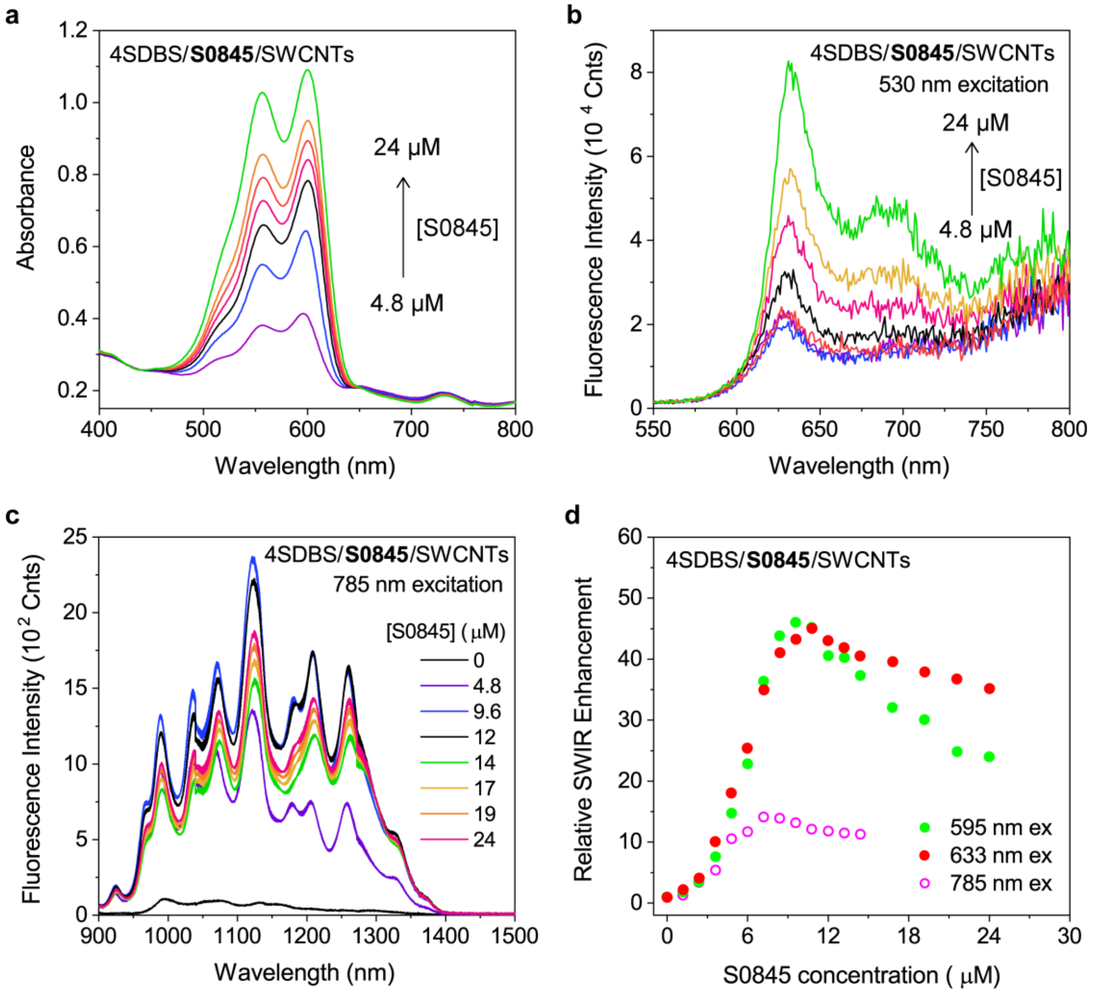

Figure S2. (a) Absorption spectra of S0845-SWCNTs complex at varied concentrations of S0845 (4.8, 9.6, 12, 14, 17, 19, 24 μM) in the presence of 4SDBS-stabilized HiPco SWCNTs. (b) Corresponding fluorescence spectra of S0845 at varied concentrations in the presence of 4SDBS-stabilized HiPco SWCNTs. The spectra were recorded upon excitation at 530 nm. (c) SWIR photoluminescence of the S0845-SWCNTs complex at varied concentrations of S0845. The spectra were recorded upon excitation at 785 nm. (d) Relative fluorescence intensities of SWCNTs at varied S0845 concentrations excited at 595 nm (green), 633 nm (red), and 785 nm (magenta).

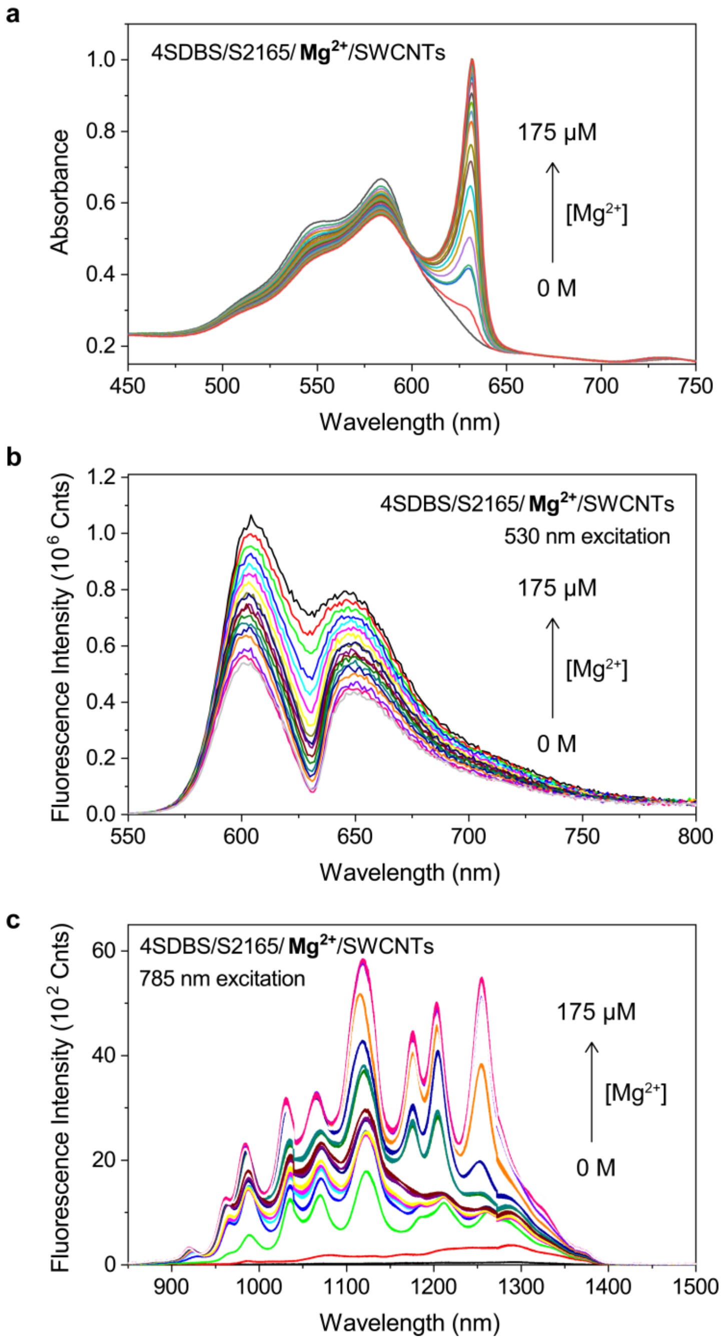

Figure S3. (a) Absorption spectra of S2165 in the presence of 4SDBS-stabilized HiPco SWCNTs at varied concentrations of $MgCl_2$. (b) Fluorescence spectra of S2165 in the presence of 4SDBS-stabilized HiPco SWCNTs at varied concentrations of $MgCl_2$ upon excitation at 530 nm. (c) SWIR photoluminescence spectra of the S2165-SWCNTs complex at varied concentrations of $MgCl_2$ upon excitation at 785 nm.

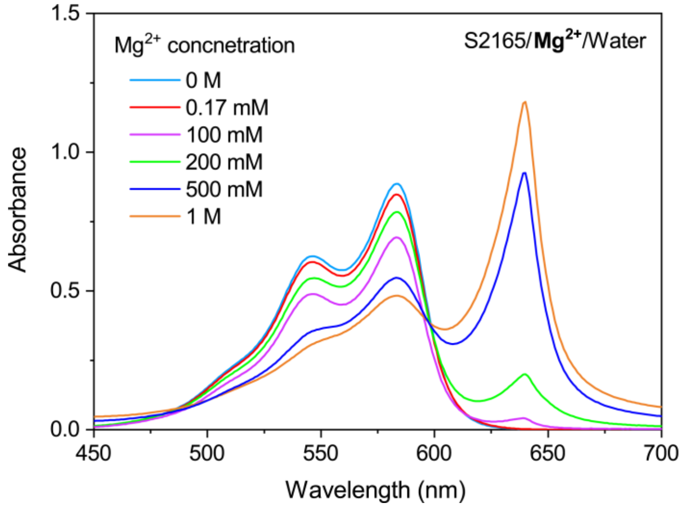

Figure S4. Absorption spectra of S2165 in water (light blue) and in the presence of $MgCl_2$ at the concentrations of 0.17 mM (concentration applied for S2165 J-aggregates-SWCNTs complex formation), 0.1 M, 0.2 M, 0.5 M, and 1.0 M.

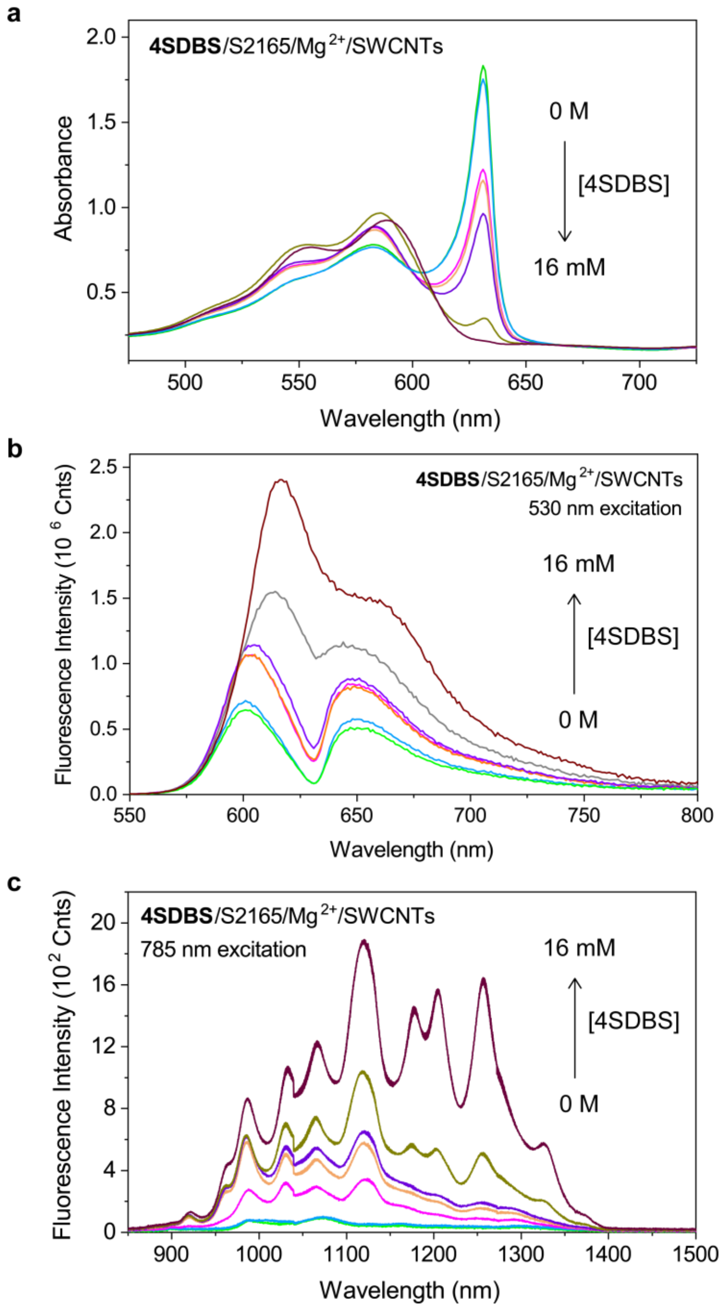

Figure S5. Effect of the 4SDBS concentration on the spectral behavior of S2165-SWCNTs complex. (a) Absorption spectra of S2165-SWCNTs complex at varied 4SDBS concentrations. (b) Fluorescence spectra of the S2165-SWCNTs complex at varied 4SDBS concentrations upon excitation at 530 nm. (c) SWIR photoluminescence spectra of S2165-SWCNTs complex at varied 4SDBS concentrations upon excitation at 785 nm. Concentration of Mg^{2+} = 0.17 mM.

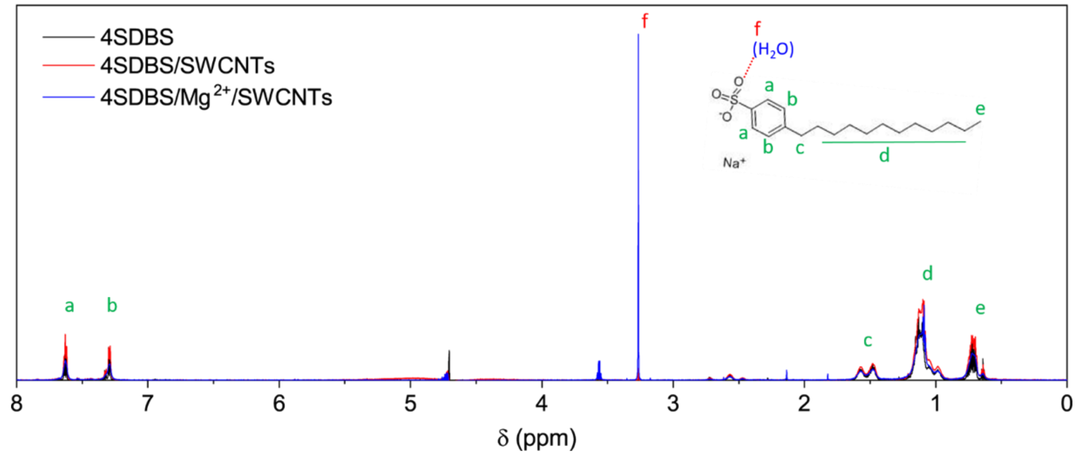

Figure S6. ^{1}H NMR spectra of free 4SDBS in water (black) and 4SDBS adsorbed on the surface of HiPco SWCNTs in the absence (red) and presence (blue) of Mg^{2+} (0.17 mM).

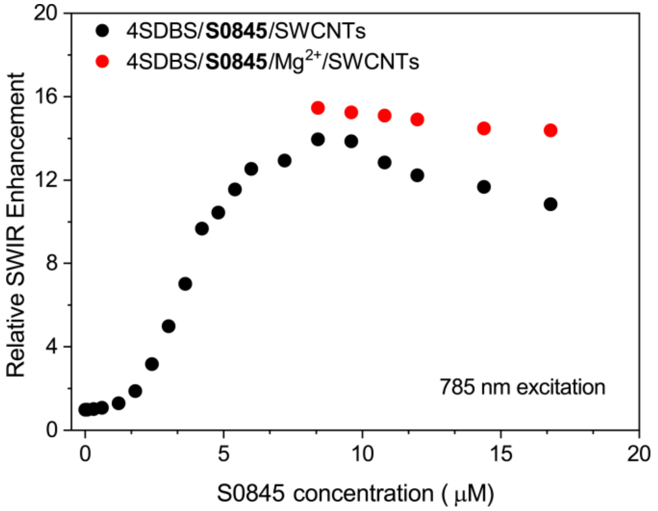

Figure S7. Relative SWIR photoluminescence enhancement of S0845-4SDBS-stabilized HiPco SWCNTs complex at varied concentrations of S0845 (black) and relative SWIR photoluminescence enhancement of the same S0845-SWCNTs complex after adding Mg^{2+} (87.5 μM) to the suspension (red). The SWIR photoluminescence intensities were measured upon 785 nm excitation.

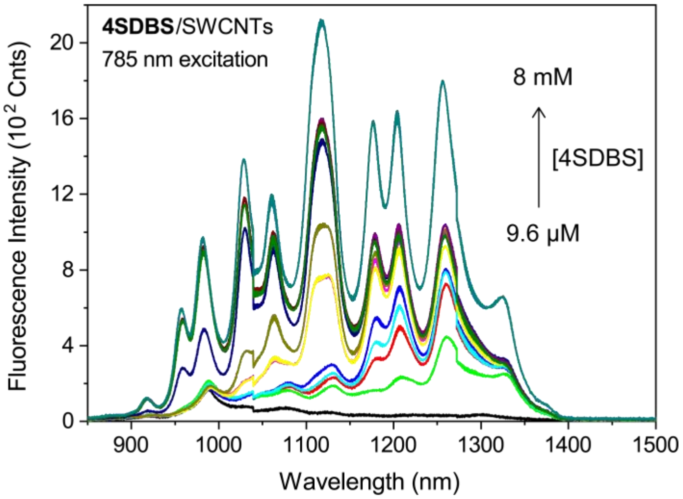

Figure S8. Spectrally resolved curvature-dependent enhancement in the SWIR photoluminescence of HiPco SWCNTs at varied 4SDBS concentrations. The photoluminescence spectra were recorded upon 785 nm excitation.

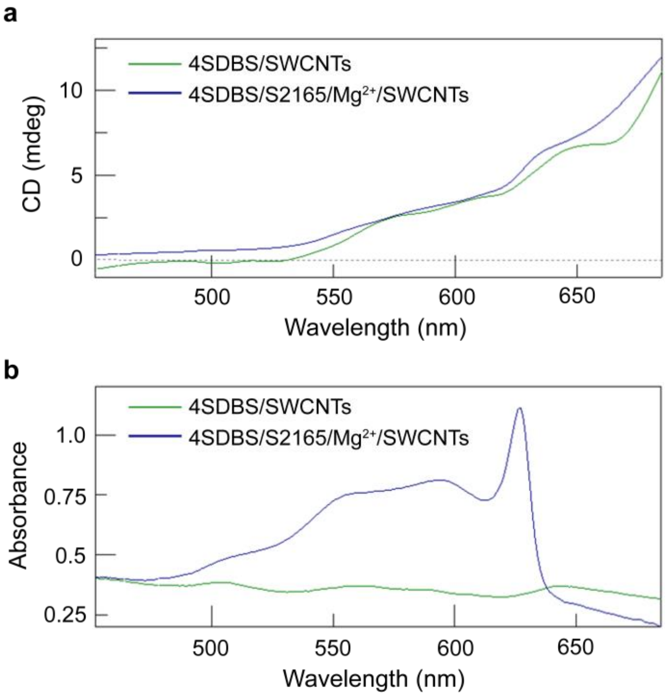

Figure S9. (a) Circular dichroism spectra and (b) absorption spectra of 4SDBS-stabilized SWCNTs dispersion (green) and the S2165 J-aggregates-SWCNTs complex (dark blue). Concentration of Mg^{2+} = 0.17 mM.

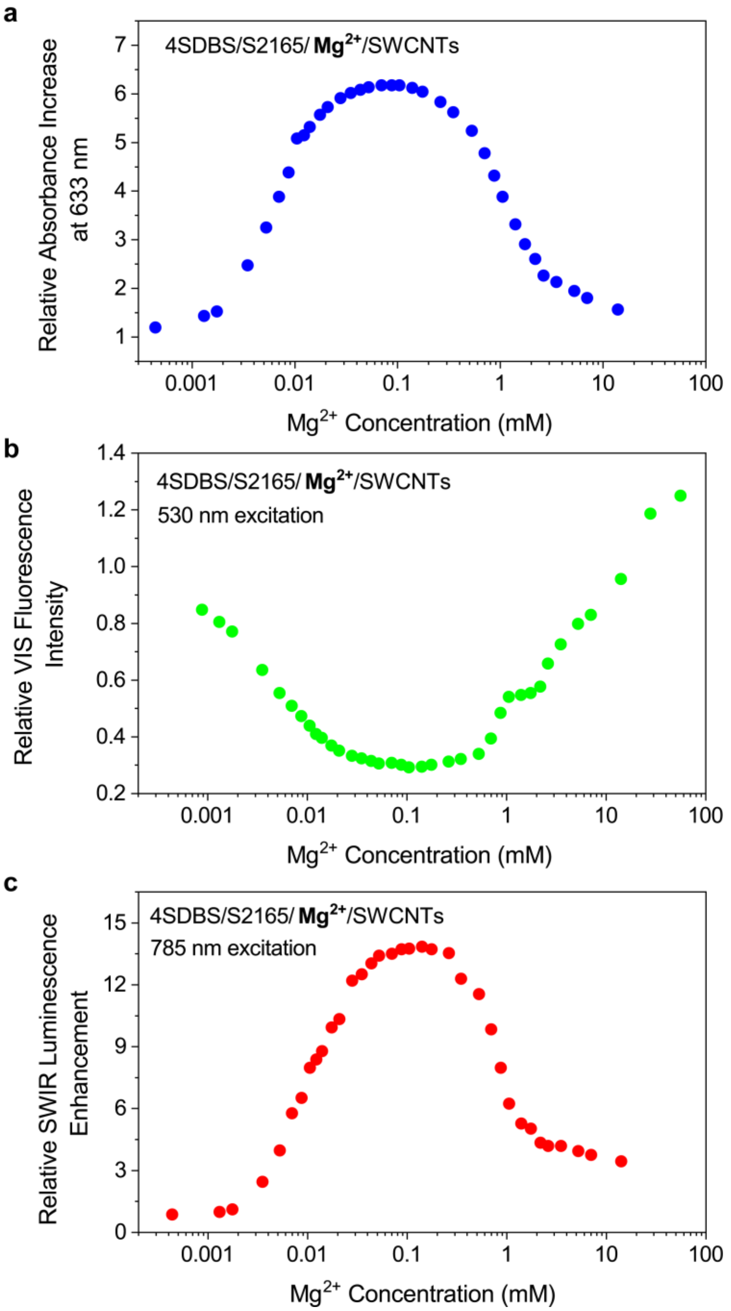

Figure S10. Titration experiments showing the effect of Mg^{2+} concentrations on the (a) relative absorbance of S2165-SWCNTs complex at 633 nm (peak absorption of S2165 J-aggregates), (b) relative fluorescence intensity of S2165-SWCNTs complex excited at 530 nm, and (c) relative SWIR photoluminescence intensity of S2165-SWCNTs complex excited at 785 nm.

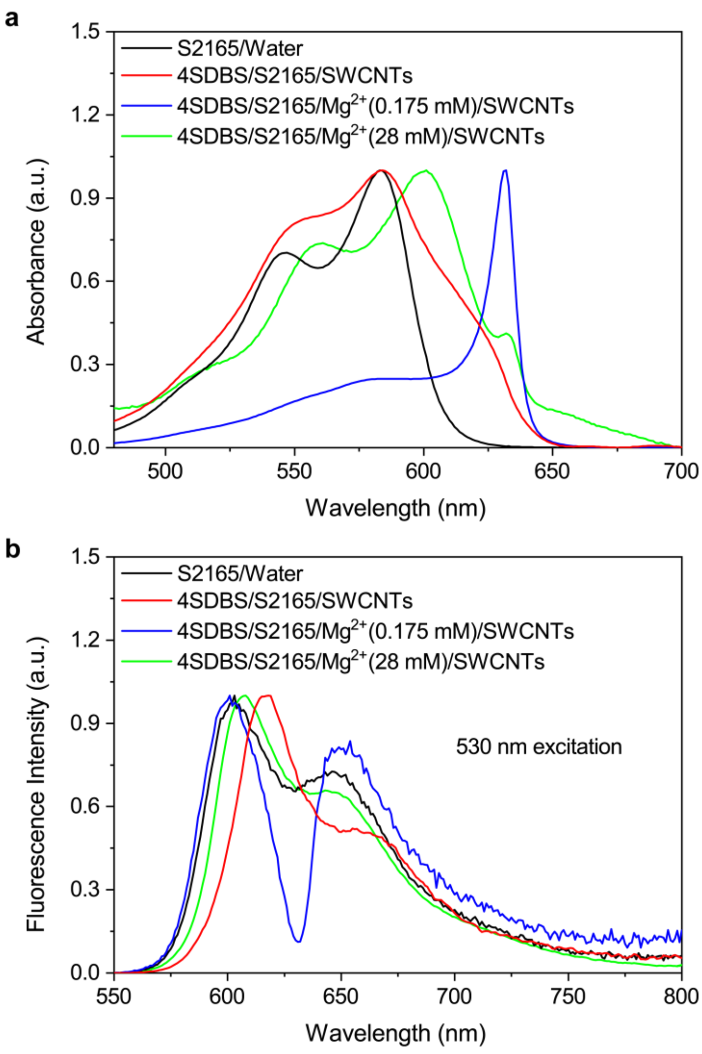

Figure S11. Absorption (a) and fluorescence (b) spectra of S2165 in water (violet), in the presence of 4SDBS-stabilized HiPco SWCNTs (black), in the presence of 4SDBS-stabilized HiPco SWCNTs after adding Mg^{2+} (175 μM, red), and in the presence of 4SDBS-stabilized HiPco SWCNTs after adding an excess of Mg^{2+} (28 mM, blue). The fluorescence spectra were recorded upon 530 nm excitation.

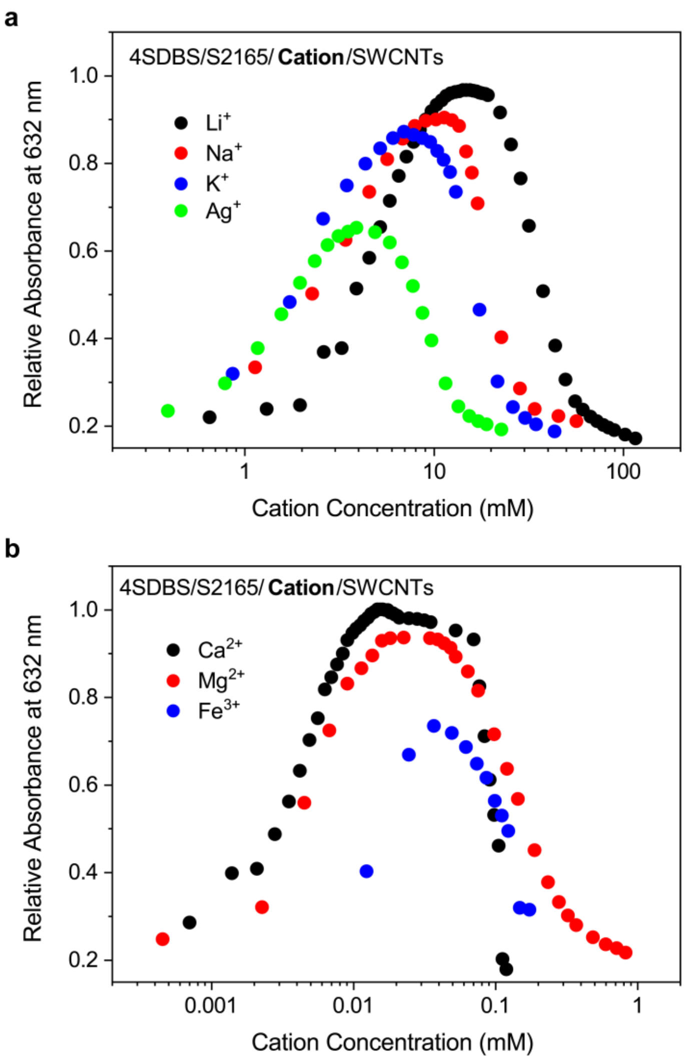

Figure S12. Titration experiments showing the effect of the cation concentration on the relative absorbance of S2165-SWCNTs complex at 632 nm (peak absorption of S2165 J-aggregates). (a) Effect of the size of cations. (b) Effect of the valence of cations.

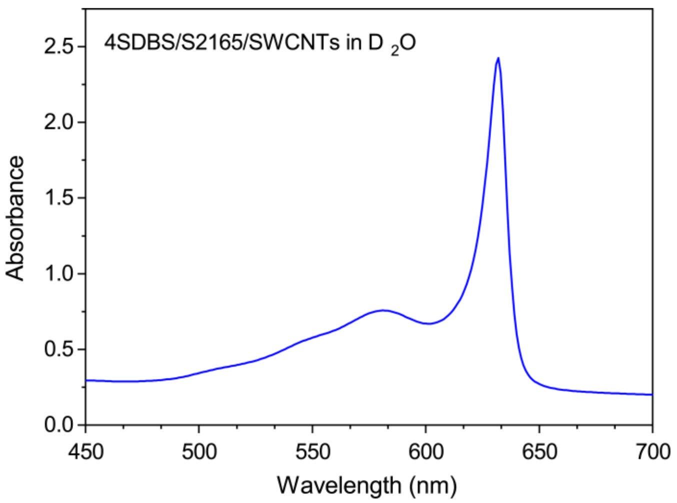

Figure S13. Absorption spectra of S2165 mixed with 4SDBS-stabilized HiPco SWCNTs dispersed in D_2O.

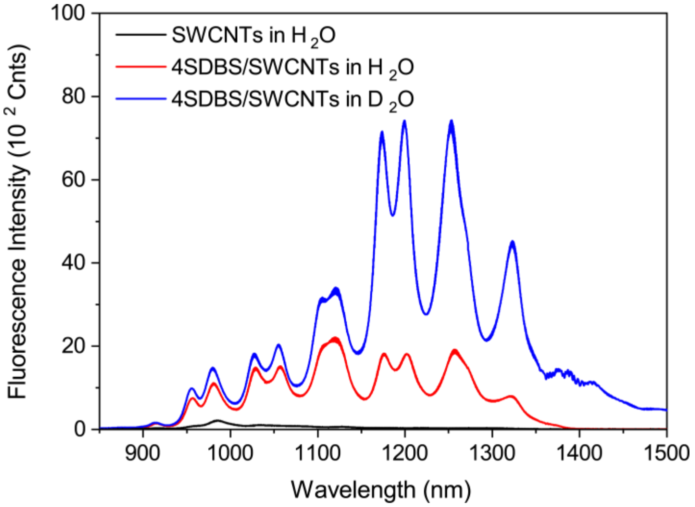

Figure S14. SWIR photoluminescence spectra of HiPco SWCNTs dispersed in water (blue), 4SDBS-stabilized HiPco SWCNTs in water (black), and 4SDBS-stabilized HiPco SWCNTs in D_2O (red) recorded upon excitation at 785 nm.

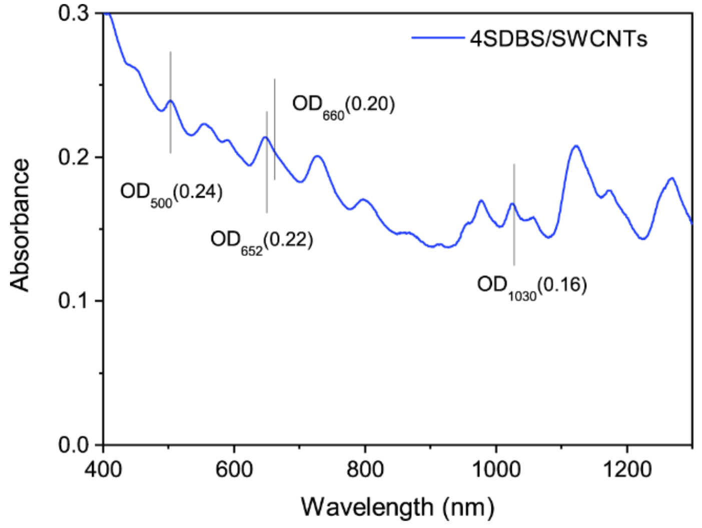

Figure S15. VIS-NIR absorption spectra of the 4SDBS dispersed SWNTs sample.

References

(1) Koh, B.; Cheng, W. Mechanisms of Carbon Nanotube Aggregation and the Reversion of Carbon Nanotube Aggregates in Aqueous Medium. Langmuir 2014, 30 (36), 10899-10909. DOI: 10.1021/la5014279.

(2) Weisman, R. B.; Bachilo, S. M. Dependence of Optical Transition Energies on Structure for Single-Walled Carbon Nanotubes in Aqueous Suspension: An Empirical Kataura Plot. Nano Letters 2003, 3 (9), 1235-1238. DOI: 10.1021/nl034428i.

(3) Wei, X.; Tanaka, T.; Li, S.; Tsuzuki, M.; Wang, G.; Yao, Z.; Li, L.; Yomogida, Y.; Hirano, A.; Liu, H.; et al. Photoluminescence Quantum Yield of Single-Wall Carbon Nanotubes Corrected for the Photon Reabsorption Effect. Nano Letters 2020, 20 (1), 410-417. DOI: 10.1021/acs.nanolett.9b04095.

(4) Tsyboulski, D. A.; Rocha, J.-D. R.; Bachilo, S. M.; Cognet, L.; Weisman, R. B. Structure-Dependent Fluorescence Efficiencies of Individual Single-Walled Carbon Nanotubes. Nano Letters 2007, 7 (10), 3080-3085. DOI: 10.1021/nl071561s.

(5) Li, J.; Jia, G.; Zhang, Y.; Chen, Y. Bond-Curvature Effect of Sidewall [2+1] Cycloadditions of Single-Walled Carbon Nanotubes: A New Criterion To the Adduct Structures. Chemistry of Materials 2006, 18 (15), 3579-3584. DOI: 10.1021/cm060563v.

(6) Yomogida, Y.; Tanaka, T.; Tsuzuki, M.; Wei, X.; Kataura, H. Automatic Sorting of Single-Chirality Single-Wall Carbon Nanotubes Using Hydrophobic Cholates: Implications for Multicolor Near-Infrared Optical Technologies. ACS Applied Nano Materials 2020, 3 (11), 11289-11297. DOI: 10.1021/acsanm.0c02389.

(7) Umadevi, D.; Sastry, G. N. Quantum Mechanical Study of Physisorption of Nucleobases on Carbon Materials: Graphene versus Carbon Nanotubes. The Journal of Physical Chemistry Letters 2011, 2 (13), 1572-1576. DOI: 10.1021/jz200705w.

(8) Choi, C.; Yun, T. G.; Hwang, B. Dispersion Stability of Carbon Nanotubes and Their Impact on Energy Storage Devices. Inorganics 2023, 11 (10), 383.

(9) Huang, W.; Wang, Z.; Luo, J. Molecular Dynamics Study of the Curvature-Driven Interactions between Carbon-Based Nanoparticles and Amino Acids. Molecules 2023, 28 (2), 482.

(10) Umadevi, D.; Sastry, G. N. Impact of the Chirality and Curvature of Carbon Nanostructures on Their Interaction with Aromatics and Amino Acids. ChemPhysChem 2013, 14 (11), 2570-2578. DOI: https://doi.org/10.1002/cphc.201300089.

(11) Gu, Z.; Yang, Z.; Chong, Y.; Ge, C.; Weber, J. K.; Bell, D. R.; Zhou, R. Surface Curvature Relation to Protein Adsorption for Carbon-based Nanomaterials. Scientific Reports 2015, 5 (1), 10886. DOI: 10.1038/srep10886.

(12) Liu, H.; Nishide, D.; Tanaka, T.; Kataura, H. Large-scale single-chirality separation of single-wall carbon nanotubes by simple gel chromatography. Nature Communications 2011, 2 (1), 309. DOI: 10.1038/ncomms1313.

(13) Gottlieb, H. E.; Kotlyar, V.; Nudelman, A. NMR Chemical Shifts of Common Laboratory Solvents as Trace Impurities. The Journal of Organic Chemistry 1997, 62 (21), 7512-7515. DOI: 10.1021/jo971176v.

(14) Kang, E.; Park, H. R.; Yoon, J.; Yu, H.-Y.; Chang, S.-K.; Kim, B.; Choi, K.; Ahn, S. A simple method to determine the water content in organic solvents using the 1H NMR chemical

shifts differences between water and solvent. Microchemical Journal 2018, 138, 395-400. DOI: https://doi.org/10.1016/j.microc.2018.01.034.

(15) Buntkowsky, G.; Breitzke, H.; Adamczyk, A.; Roelofs, F.; Emmler, T.; Gedat, E.; Grünberg, B.; Xu, Y.; Limbach, H.-H.; Shenderovich, I.; et al. Structural and dynamical properties of guest molecules confined in mesoporous silica materials revealed by NMR. Physical Chemistry Chemical Physics 2007, 9 (35), 4843-4853, 10.1039/B707322D. DOI: 10.1039/B707322D.

(16) Urbic, T. Ions increase strength of hydrogen bond in water. Chemical Physics Letters 2014, 610-611, 159-162. DOI: https://doi.org/10.1016/j.cplett.2014.06.054.

(17) Yan, H.; Yuan, S.-L.; Xu, G.-Y.; Liu, C.-B. Effect of Ca2+ and Mg2+ Ions on Surfactant Solutions Investigated by Molecular Dynamics Simulation. Langmuir 2010, 26 (13), 10448-10459. DOI: 10.1021/la100310w.

(18) Al Bawab, A.; Bozeya, A.; Odeh, F. Effect of Surfactant Tail Structure on Phase Behavior of Branched and Linear Alkylbenzene Sulfonate in Water and Oil Ternary Systems. GSTF Journal of Chemical Sciences (JChem) 2014, 1 (2), 5. DOI: 10.7603/s40837-014-0005-5.

(19) Zhao, T.; Xu, G.; Yuan, S.; Chen, Y.; Yan, H. Molecular Dynamics Study of Alkyl Benzene Sulfonate at Air/Water Interface: Effect of Inorganic Salts. The Journal of Physical Chemistry B 2010, 114 (15), 5025-5033. DOI: 10.1021/jp907438x.

(20) Jeffrey, G. A. An Introduction to Hydrogen Bonding; Oxford University Press, 1997.

(21) W, J. O. T. Hydrogen bonding: S.N. Vinogradov and R.H. Linnell, Van Nostrand Reinhold Company, London, 1971, pp. 319, price £4.75. Journal of Molecular Structure 1972, 14 (3), 470. DOI: https://doi.org/10.1016/0022-2860(72)85199-8.

(22) Stryer, L. Excited-State Proton-Transfer Reactions. A Deuterium Isotope Effect on Fluorescence. Journal of the American Chemical Society 1966, 88 (24), 5708-5712. DOI: 10.1021/ja00976a004.

(23) Fürstenberg, A. Water in Biomolecular Fluorescence Spectroscopy and Imaging: Side Effects and Remedies. CHIMIA 2017, 71 (1-2), 26. DOI: 10.2533/chimia.2017.26 (acccessed 2023/09/12).

(24) Klehs, K.; Spahn, C.; Endesfelder, U.; Lee, S. F.; Fürstenberg, A.; Heilemann, M. Increasing the Brightness of Cyanine Fluorophores for Single-Molecule and Superresolution Imaging. ChemPhysChem 2014, 15 (4), 637-641. DOI: https://doi.org/10.1002/cphc.201300874.

(25) Maillard, J.; Klehs, K.; Rumble, C.; Vauthey, E.; Heilemann, M.; Fürstenberg, A. Universal quenching of common fluorescent probes by water and alcohols. Chemical Science 2021, 12 (4), 1352-1362, 10.1039/D0SC05431C. DOI: 10.1039/D0SC05431C.

(26) Moore, V. C.; Strano, M. S.; Haroz, E. H.; Hauge, R. H.; Smalley, R. E.; Schmidt, J.; Talmon, Y. Individually suspended single-walled carbon nanotubes in various surfactants. Nano Letters 2003, 3 (10), 1379-1382, Article. DOI: 10.1021/nl034524j.

(27) Landi, B. J.; Ruf, H. J.; Worman, J. J.; Raffaelle, R. P. Effects of alkyl amide solvents on the dispersion of single-wall carbon nanotubes. J. Phys. Chem. B 2004, 108 (44), 17089-17095, Article. DOI: 10.1021/jp047521j.

(28) Bahr, J. L.; Mickelson, E. T.; Bronikowski, M. J.; Smalley, R. E.; Tour, J. M. Dissolution of small diameter single-wall carbon nanotubes in organic solvents? Chemical Communications 2001, (2), 193-194, Article. DOI: 10.1039/b008042j.

(29) Ivanova, M. V.; Lamprecht, C.; Loureiro, M. J.; Huzil, J. T.; Foldvari, M. Pharmaceutical characterization of solid and dispersed carbon nanotubes as nanoexcipients. Int. J. Nanomed. 2012, 7, 403-415, Article. DOI: 10.2147/ijn.S27442.

(30) Kang, Y. J.; Taton, T. A. Micelle-encapsulated carbon nanotubes: A route to nanotube composites. Journal of the American Chemical Society 2003, 125 (19), 5650-5651, Article. DOI: 10.1021/ja034082d.

(31) Ikeda, A.; Hamano, T.; Hayashi, K.; Kikuchi, J. Water-solubilization of nucleotides-coated single-walled carbon nanotubes using a high-speed vibration milling technique. Org. Lett. 2006, 8 (6), 1153-1156, Article. DOI: 10.1021/ol053089s.

(32) Rastogi, R.; Kaushal, R.; Tripathi, S. K.; Sharma, A. L.; Kaur, I.; Bharadwaj, L. M. Comparative study of carbon nanotube dispersion using surfactants. J. Colloid Interface Sci. 2008, 328 (2), 421-428, Article. DOI: 10.1016/j.jcis.2008.09.015.

(33) Backes, C.; Mundloch, U.; Ebel, A.; Hauke, F.; Hirsch, A. Dispersion of HiPco® and CoMoCAT® Single-Walled Nanotubes (SWNTs) by Water Soluble Pyrene Derivatives-Depletion of Small Diameter SWNTs. Chem.-Eur. J. 2010, 16 (11), 3314-3317, Article. DOI: 10.1002/chem.200903420.

(34) Streit, J. K.; Bachilo, S. M.; Ghosh, S.; Lin, C. W.; Weisman, R. B. Directly Measured Optical Absorption Cross Sections for Structure-Selected Single-Walled Carbon Nanotubes. Nano Letters 2014, 14 (3), 1530-1536, Article. DOI: 10.1021/nl404791y.
